# The oncogenic role of human papillomavirus in breast cancer: a comprehensive systematic review and meta-analysis

**DOI:** 10.3389/fmicb.2025.1712118

**Published:** 2025-11-14

**Authors:** Dana Koishybayeva, Saule Balmagambetova, Bazylbek Zhakiev, Arip Koishybayev, Nadiar M. Mussin, Svetlana Sakhanova, Marzhan Aitmagambetova, Anar Tulyayeva, Amin Tamadon

**Affiliations:** 1 Department of Surgical Diseases-2, West Kazakhstan Marat Ospanov Medical University, Aktobe, Kazakhstan; 2 Department of Oncology, West Kazakhstan Marat Ospanov Medical University, Aktobe, Kazakhstan; 3 Department of General Surgery, West Kazakhstan Marat Ospanov Medical University, Aktobe, Kazakhstan; 4 Scientific and Practical Center, West Kazakhstan Marat Ospanov Medical University, Aktobe, Kazakhstan; 5 Department of Natural Sciences, West Kazakhstan Marat Ospanov Medical University, Aktobe, Kazakhstan

**Keywords:** human papillomaviruses, breast neoplasms, meta-analysis, case–control studies, systematic review

## Abstract

**Introduction:**

Human papillomavirus (HPV) has been increasingly implicated in the pathogenesis of breast cancer (BC), though its role remains controversial. Understanding HPV prevalence and genotype distribution across histological types and regions may clarify this potential association.

**Methods:**

A comprehensive systematic review and meta-analysis was conducted using PubMed, Scopus, and Web of Science databases for studies published between January 1990 and April 2025. Eligible studies reported HPV prevalence in BC tissues stratified by histological classification. Non-English studies, reviews, and those lacking histological stratification were excluded. Data from 49 studies encompassing 4,173 BC cases were extracted. Pooled HPV prevalence and odds ratios (ORs) were calculated using random-effects models. Subgroup analyses were performed by histology, geographic region, and HPV genotype (16/18). Risk of bias was assessed using the Joanna Briggs Institute (JBI) checklist for cross-sectional studies and the Newcastle–Ottawa Scale for case-control designs.

**Results:**

The pooled prevalence of HPV in BC tissues was 23% (95% CI: 18–28%), highest in invasive ductal carcinoma (24%). HPV-positive individuals exhibited a 3.6-fold higher risk of developing BC (OR = 3.63, 95% CI: 2.33–5.64), with the strongest association in invasive lobular carcinoma (OR = 4.41). HPV-18 showed a more consistent correlation with BC than HPV-16. Regional variation was observed, with Asian populations showing higher HPV prevalence and stronger associations.

**Discussion:**

This meta-analysis suggests a significant association between HPV infection—particularly genotype 18—and breast cancer risk, especially in Asian regions and specific histological subtypes. These findings highlight the need for mechanistic studies and standardized molecular detection to elucidate the potential oncogenic role of HPV in breast tissue.

**Systematic review registration:**

https://www.crd.york.ac.uk/PROSPERO/display_record.php?RecordID=1051960 identifier CRD420251051960

## 1 Introduction

Breast cancer (BC) is the most frequently diagnosed malignancy and the leading cause of cancer-related death among women worldwide (Smolarz et al., 2022). BC accounts for approximately 12% of all cancer cases globally, with over 2 million new cases reported in 2020 (Sedeta et al., 2023; Lukasiewicz et al., 2021). It is characterized by significant heterogeneity in its histopathological types, epidemiological factors, and clinical outcomes. Among these types, invasive lobular carcinoma (ILC), invasive ductal carcinoma (IDC), and ductal carcinoma *in situ* (DCIS) are the most prevalent. Understanding the potential infectious etiology of BC, including viral involvement, is crucial for advancing preventive and therapeutic strategies (Liu and Yu, 2023). The prevalence of BC is increasing, particularly in regions adopting Western lifestyle behaviors, such as South America, Africa, and Asia (Sedeta et al., 2023). Human papillomavirus (HPV), a DNA virus from the papillomaviridae family, predominantly targets epithelial tissues (Hareza et al., 2022). The virus is classified into non-oncogenic low-risk and oncogenic high-risk categories. Low-risk strains of HPV are typically responsible for the development of genital warts, whereas high-risk strains—particularly HPV types 16 and 18—are linked to the onset of cancers affecting the cervix, vulva, vagina, anus, penis, and the oropharyngeal region (Siddiqi and Ridker, 2019; Pesut et al., 2021). HPV’s oncogenic potential is primarily due to the E6 and E7 oncoproteins, which interfere with tumor suppressor proteins like p53 and pRB, promoting cell cycle disruption and carcinogenesis (Hareza et al., 2022).

HPV is primarily transmitted through sexual contact and, less commonly, via vertical transmission during childbirth (Malagon et al., 2021; Wierzbicka et al., 2023; Khayargoli et al., 2023; Freitas et al., 2013). Although these routes are well established, potential dissemination to mammary tissue remains hypothetical. Proposed mechanisms include hematogenous or lymphatic spread from other infected sites and retrograde ductal migration through the nipple–areolar complex (Lawson et al., 2015; Bodaghi et al., 2005; Akil et al., 2008; de Villiers et al., 2005; Purrahman et al., 2022; Blanco et al., 2021). While biologically plausible, direct evidence remains limited. Detections of HPV DNA and E6/E7 transcripts in nipple–areolar ducts and reports of circulating HPV DNA support biological plausibility for mammary tissue exposure.

The prospective correlation between HPV and breast carcinoma has emerged as a topic of significant scholarly discourse and investigation. While some studies suggest a possible link, others find no significant association (Purrahman et al., 2022). Previous studies investigating the occurrence of HPV DNA within BC tissues have reported widely varying rates, influenced by factors such as geographic region, population characteristics, histological subtype, and diagnostic methods. HPV types 16 and 18, which are recognized for their strong cancer-causing potential in cervical malignancies, have also been frequently identified in BC tissues. Nevertheless, their definitive role in the initiation or progression of BC is still uncertain.

To explore a possible causal relationship between HPV infection and BC development, we conducted an extensive systematic review and meta-analysis following the 2020 PRISMA (Preferred Reporting Items for Systematic Reviews and Meta-Analyses) guidelines. We aimed to evaluate the prevalence of HPV DNA in breast tumors quantitatively, explore associations with specific histological types (IDC, ILC, DCIS), and analyze trends in geographical and genotype-specific distribution. To our knowledge, this paper represents the first comprehensive meta-analysis that systematically categorizes findings by histopathological classification, enabling a more nuanced understanding of HPV’s potential oncogenic mechanisms across distinct BC phenotypes. Our work extends prior syntheses—including the updated meta-analysis by Awan et al. (2023)—by stratifying results *a priori* by histopathology (IDC, ILC, DCIS), geography, and genotype, and by formally exploring method-related heterogeneity. Compared with the recent comprehensive meta-analysis by Awan et al. (2023), the present review provides additional resolution by analyzing HPV prevalence and risk stratified by histological subtype (IDC, ILC, DCIS) and geographic region, incorporating studies published through 2025. The synthesized evidence may significantly contribute to the ongoing discourse regarding viral oncogenesis in BC and could inform the development of targeted screening protocols and therapeutic interventions.

## 2 Materials and methods

### 2.1 Search strategy

This thorough and systematic review of existing literature identified relevant studies that investigate the presence of HPV infection in patients diagnosed with BC. The study was registered in PROSPERO on May 19, 2025 (CRD420251051960). The protocol was registered retrospectively in PROSPERO after the database search was completed; no deviations from the initial search or analysis plan occurred. The search covered publications from January 1990 to April 2025 across three major databases: PubMed, Scopus, and Web of Science. A combination of controlled vocabulary (MeSH terms) and free-text keywords was used to maximize the sensitivity of the search strategy (Table 1). Search terms and selection criteria were harmonized with, but broadened beyond, prior frameworks (Awan et al., 2023), to capture histotype-specific reporting.

**Table 1:** Search strategy across databases for human papillomavirus (HPV) and breast cancer meta-analysis.

No.	Queries
#1	“HPV” OR “human papillomavirus viruses” OR “papillomavirus” OR “papillomaviridae”
#2	“neoplasms” OR “neoplasm” OR “cancer”
#3	“breast neoplasms” OR “breast” OR “breast neoplasms” OR “breasts” OR “breast neoplasm” OR “breast cancer”
#4	“tissues” OR “tissue”
#5	#1 AND #2 AND #3 AND #4

### 2.2 Selection of studies

Well-defined inclusion and exclusion criteria were delineated to guarantee the pertinence and integrity of the chosen studies. Inclusion criteria encompassed studies reporting HPV prevalence in BC tissues, stratified by histological types (IDC, ILC, and DCIS). Eligible studies included those with cross-sectional, case-based, or prevalence-focused designs. Research studies were excluded from consideration if they fulfilled any of the subsequent criteria: published in languages distinct from English; classified as letters, commentaries, reviews, case series, editorials, or commission reports; recognized as duplicate publications; did not report the raw data necessary to compute HPV prevalence or odds ratios (OR)—specifically, the number of BC and control samples and the counts of HPV-positive cases; or did not offer stratification based on histological tumor type. These stringent criteria were implemented to enhance methodological rigor, ensure data comparability across studies, and allow stratified analyses by histological types. Only English-language studies were included to ensure methodological consistency; we acknowledge this may introduce language bias, particularly for Asia and South America.

### 2.3 Data extraction

**Figure 1:**
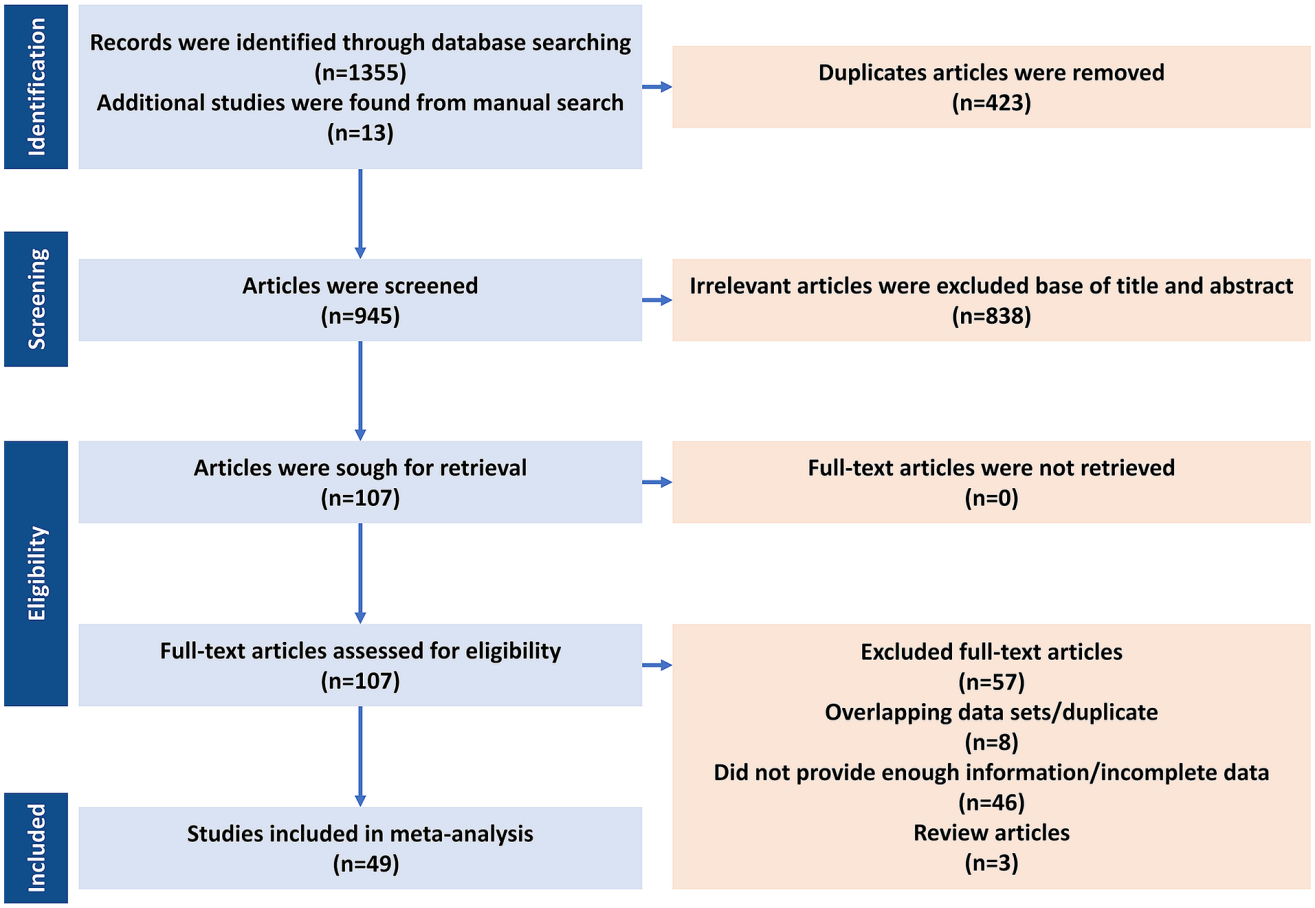
PRISMA flow diagram for the systematic search and article selection process (*n* = 49; 4,173 BC cases) for human papillomavirus (HPV) and breast cancer meta-analysis.

After the initial screening, three independent reviewers (AT, SB, and DK) evaluated the titles and abstracts of the studies that were identified, proceeding to full-text review when necessary. Throughout the search, 1,368 articles were screened, of which 49 (4,173 BC cases) met the eligibility criteria and were included in the final meta-analysis (Table 2—Summary table of studies reporting the presence of HPV in BC patients from 1992 to 2022) (Lawson et al., 2015; Akil et al., 2008; de Villiers et al., 2005; Hennig et al., 1999; Yu et al., 2000; Damin et al., 2004; Widschwendter et al., 2004; Kroupis et al., 2006; Choi et al., 2007; Duo et al., 2008; Khan et al., 2008; Heng et al., 2009; Mendizabal-Ruiz et al., 2009; Aguayo et al., 2011; Nascimento et al., 2024; Belachew et al., 2024; Mareti et al., 2023; Maldonado-Rodriguez et al., 2022; Alinezhadi et al., 2022; Gupta et al., 2021; Elagali et al., 2021; Tawfeik et al., 2020; Sher et al., 2020; Khodabandehlou et al., 2019; Salman et al., 2017; Balci et al., 2019; Antonsson et al., 2011; Herrera-Goepfert et al., 2011; Baltzell et al., 2012; Frega et al., 2012; Habyarimana et al., 2018; Ghaffari et al., 2018; Wang et al., 2017; Ngamkham et al., 2017; Naushad et al., 2017; Glenn et al., 2012; Islam et al., 2017; Doosti et al., 2016; Sigaroodi et al., 2012; Li et al., 2015; Gannon et al., 2015; Herrera-Goepfert et al., 2013; Liang et al., 2013; Pereira Suarez et al., 2013; Ahangar-Oskouee et al., 2014; Fu et al., 2015; Fernandes et al., 2015; Manzouri et al., 2014; Hong and Tang, 2014). Key variables identified comprised the initial author’s name, publication year, geographical study site, sample size, and HPV prevalence in BC cases, detected HPV genotypes, presence of coinfections, and the diagnostic methods used. Data extraction followed the PRISMA guidelines (Figure 1). The pooled HPV prevalence and odds ratios (ORs) were calculated using random-effects models. Subgroup analyses were performed by histology, region, and HPV genotype (HPV-16/18).

**Table 2:** Summary of the included studies for human papillomavirus (HPV) and breast cancer meta-analysis.

Author, year (references)	Country	Study design	HPV-positive/total (*n*/*N*)	Prevalence (%)
Aguayo et al. (2011)	Chile	Cross-sectional	4/46	8.70
Ahangar-Oskouee et al. (2014)	Iran	Case–control	22/65	33.85
Akil et al. (2008)	Syria	Cross-sectional	69/113	61.06
Alinezhadi et al. (2022)	Iran	Cross-sectional	63/95	66.32
Antonsson et al. (2011)	Australia	Cross-sectional	27/54	50.00
Balci et al. (2019)	United States	Case–control	8/18	44.44
Baltzell et al. (2012)	United States	Cross-sectional	6/70	8.57
Belachew et al. (2024)	Ethiopia	Case–control	14/66	21.21
Choi et al. (2007)	South Korea	Case–control	8/123	6.50
Damin et al. (2004)	Brazil	Case–control	25/101	24.75
de Villiers et al. (2005)	Germany	Cross-sectional	25/29	86.21
Doosti et al. (2016)	Iran	Case–control	20/87	22.99
Duo et al. (2008)	Italy	Cross-sectional	2/52	3.85
Elagali et al. (2021)	Sudan	Cross-sectional	13/150	8.67
Fernandes et al. (2015)	Venezuela	Cross-sectional	10/24	41.67
Frega et al. (2012)	Italy	Case–control	9/31	29.03
Fu et al. (2015)	China	Cross-sectional	25/169	14.79
Gannon et al. (2015)	Australia	Case–control	13/80	16.25
Ghaffari et al. (2018)	Iran	Cross-sectional	4/72	5.56
Glenn et al. (2012)	Australia	Case–control	25/50	50.00
Gupta et al. (2021)	Qatar	Case–control	48/74	64.86
Habyarimana et al. (2018)	Rwanda	Cross-sectional	22/47	46.81
Heng et al. (2009)	Australia	Case–control	8/26	30.77
Hennig et al. (1999)	Norway	Cross-sectional	19/41	46.34
Herrera-Goepfert et al. (2011)	Mexico	Cross-sectional	17/70	24.29
Herrera-Goepfert et al. (2013)	Mexico	Cross-sectional	8/20	40.00
Hong and Tang (2014)	China	Case–control	23/45	51.11
Islam et al. (2017)	India	Case–control	203/313	64.86
Khan et al. (2008)	Japan	Case–control	26/124	20.97
Khodabandehlou et al. (2019)	Iran	Case–control	35/72	48.61
Kroupis et al. (2006)	Greece	Cross-sectional	17/107	15.89
Lawson et al. (2015)	Australia	Case–control	13/28	46.43
Li et al. (2015)	China	Case–control	3/187	1.60
Liang et al. (2013)	China	Case–control	48/224	21.43
Maldonado-Rodriguez et al. (2022)	Mexico	Case–control	12/59	20.34
Manzouri et al. (2014)	Iran	Case–control	10/55	18.18
Mareti et al. (2023)	Greece	Case–control	7/57	12.28
Mendizabal-Ruiz et al. (2009)	Mexico	Case–control	3/67	4.48
Nascimento et al. (2024)	Brazil	Cross-sectional	20/56	35.71
Naushad et al. (2017)	Pakistan	Cross-sectional	45/250	18.00
Ngamkham et al. (2017)	Thailand	Case–control	15/350	4.29
Pereira Suarez et al. (2013)	Argentina	Cross-sectional	16/61	26.23
Salman et al. (2017)	United Kingdom	Case–control	35/72	48.61
Sher et al. (2020)	Qatar	Case–control	10/50	20.00
Sigaroodi et al. (2012)	Iran	Case–control	15/79	18.99
Tawfeik et al. (2020)	Egypt	Case–control	4/20	20.00
Wang et al. (2017)	China	Cross-sectional	14/81	17.28
Widschwendter et al. (2004)	Austria	Cross-sectional	7/11	63.64
Yu et al. (2000)	China	Cross-sectional	18/32	56.25

Prevalence values are shown only for descriptive purposes. All statistical syntheses used the exact raw counts (events/total) extracted from each study.

### 2.4 Quality assessment

Three reviewers (AT, SB, and DK) independently evaluated the methodological quality of each study included in the analysis. For cross-sectional studies, they applied the Joanna Briggs Institute (JBI) Critical Appraisal Checklist, while for case–control studies, the Newcastle–Ottawa Scale was used to assess the risk of bias. To ensure consistency and methodological integrity, any discrepancies in quality assessments were addressed through discussion until a consensus was reached among the reviewers.

### 2.5 Statistical analysis

Quantitative analyses were conducted after data extraction, using Microsoft Excel for preliminary organization and RevMan (version 5.4) together with R version (4.5.0) for advanced meta-analytic procedures. Odds ratios represented the odds of detecting HPV DNA in breast-cancer tissues compared with non-cancerous control breast tissues. Various R packages were utilized for the meta-analyses. The prevalence of HPV infection was estimated using the binomial distribution formula, with corresponding standard errors (SE). To accommodate the anticipated variability among studies, a random-effects model was applied. Heterogeneity was evaluated using Cochran’s *Q* test and the *I*^2^ statistic, with *I*^2^ values of 25, 50, and 75% indicating low, moderate, and high heterogeneity, respectively. Forest plots were generated to visually depict the effect sizes (ES) along with their 95% confidence intervals (95% CI). Subgroup analyses were also conducted to investigate possible sources of heterogeneity. The certainty of evidence was not assessed using the GRADE approach. We explored heterogeneity using subgroup analyses and meta-regression (metafor, REML) with moderators: (i) detection method (e.g., consensus PCR/nested PCR/hybrid capture/RT-PCR), (ii) specimen type (FFPE vs. fresh/frozen), and (iii) geographic region. For case–control studies, log-ORs were modeled against these moderators; for prevalence, logit-transformed proportions were used.

Prevalence (one-group) and odds-ratio (case–control) analyses were conducted separately. To address potential confounding by detection method or specimen handling, subgroup and meta-regression analyses were performed with moderators for assay type (consensus PCR, nested PCR, hybrid capture, RT-PCR) and tissue source (FFPE vs. fresh/frozen). Certainty of evidence (GRADE) was not performed due to observational designs and methodological heterogeneity. Studies with zero/near-zero cells were handled using continuity corrections per standard practice; resultant wide CIs indicate imprecision.

## 3 Results

### 3.1 Characteristics of included studies

A total of 1,381 records were identified, and 49 studies (4,173 BC cases) met the inclusion criteria (Figure 1). The final meta-analysis included 49 studies investigating the presence of HPV DNA in BC tissues, comprising a total of 4,173 BC cases. The analysis was conducted in two phases: (1) a one-group proportion meta-analysis to estimate the pooled prevalence of HPV among BC patients, and (2) a case–control meta-analysis comparing HPV prevalence between cancerous and non-cancerous breast tissues. The 86% prevalence in de Villiers et al. (2005) reflects small sample size and early detection platforms; exclusion in sensitivity analysis did not materially change pooled estimates.

Detailed per-study genotype data have been moved to Supplementary Table S1. Table 3 summarizes the 10 most frequent genotypes by continent. The analysis of HPV genotype distribution across the included studies revealed considerable variation in the prevalence of individual HPV types and co-infections (Supplementary Table S1). HPV-16 was the most frequently reported genotype, appearing in numerous studies with varying case numbers, followed by HPV-18, HPV-33, and HPV-31. Several studies also identified less common genotypes such as HPV-35, HPV-66, and HPV-58, while others reported the presence of low-risk types like HPV-6 and HPV-11. Notably, co-infections involving multiple HPV types were documented in many reports, suggesting a complex pattern of viral presence in affected individuals. The total number of cases per study ranged widely, with some studies focusing on a few specific genotypes and others providing broader screening results encompassing over 20 genotypes. This variation highlights both geographic and methodological differences in HPV detection and reporting, underlining the importance of comprehensive genotyping in understanding the epidemiology of HPV infections.

**Table 3:** Most frequent high-risk human papillomavirus (HPV) genotypes in breast cancer by continent (top 10 by frequency).

Continent	Most common HPV genotypes (descending frequency)	Total cases (*n*)	% of all HPV-positive BC samples
Asia	16, 18, 33, 58, 52, 31, 35, 45, 66, 11	356	55.0%
Europe	16, 18, 33, 31, 45, 35, 6, 11, 58, 66	142	21.9%
Americas	16, 18, 33, 31, 58, 35, 52, 45, 66, 11	106	16.4%
Africa	16, 18, 33, 45, 35, 31, 58, 52, 6, 11	57	8.8%
Oceania	16, 18, 33, 31, 45, 35, 58, 6, 11, 52	29	4.5%

HPV-16 and HPV-18 remain globally predominant. Regional variation is evident for genotypes 31, 33, 35, 45, 52, 58, and 66. Detailed per-study genotype data are provided in Supplementary Table S1.

### 3.2 Methodological quality and bias risk

Quality assessment using JBI and Newcastle–Ottawa tools revealed that 85% of studies (42/49) demonstrated moderate quality (mean scores: 6/8 for cross-sectional, 6/9 for case–control) (Supplementary Tables S2, S3). Common limitations included incomplete adjustment for confounders (75% of studies) and variability in HPV detection protocols. Notably, heterogeneity was lowest for ILC (*I*^2^ = 11.6%) and DCIS (*I*^2^ = 25.9%), suggesting robust subtype-specific findings, while IDC exhibited substantial heterogeneity (*I*^2^ = 91.6%), likely reflecting methodological diversity across studies.

### 3.3 One-group proportion meta-analysis

**Figure 2:**
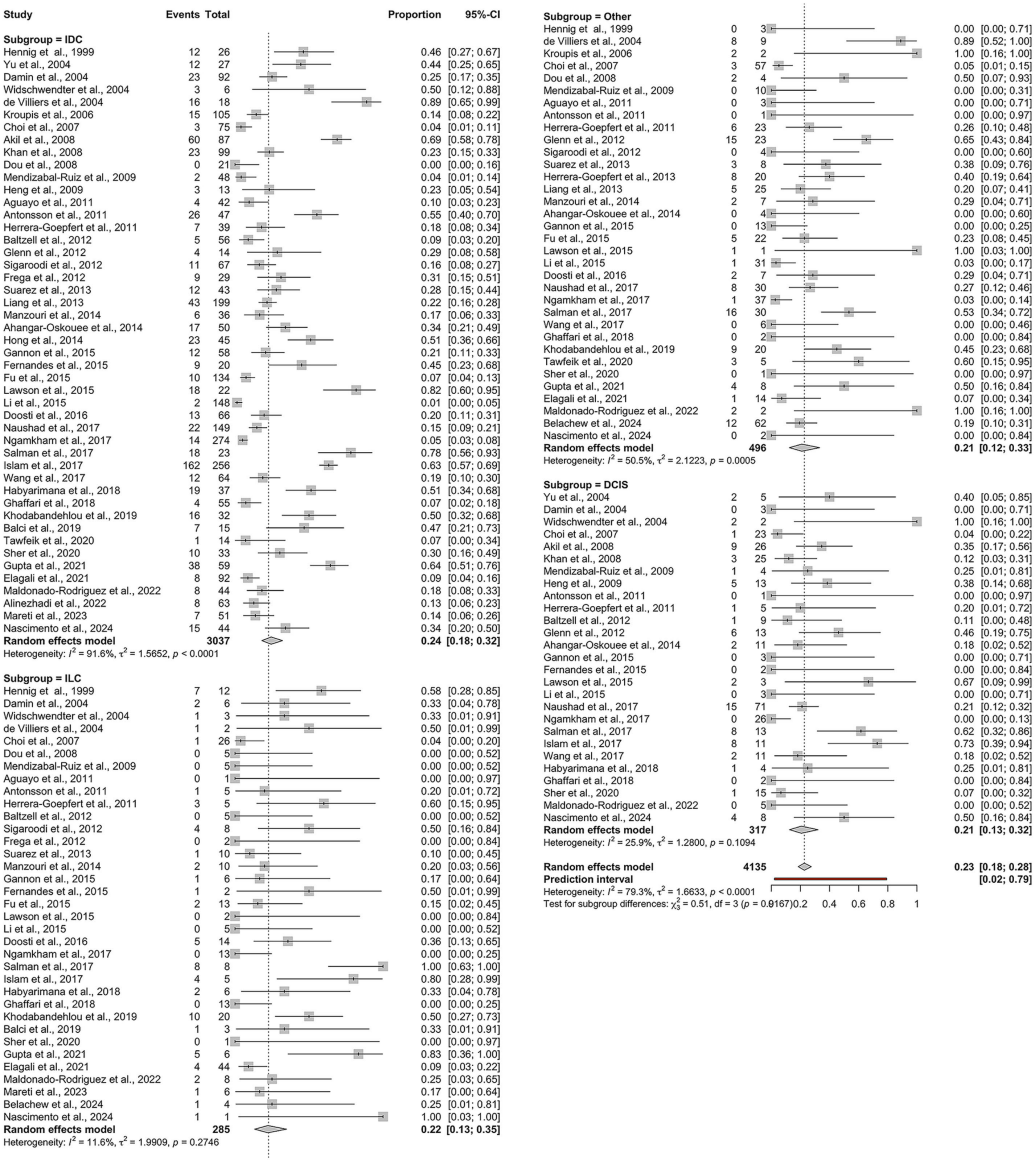
Overall pooled prevalence of human papillomavirus (HPV) across histological subtypes of breast cancer: IDC, ILC, DCIS, and other histological types.

The pooled proportion of HPV-positive BC cases across all studies was 23% (95% CI: 18–28%). Subgroup analysis based on histological type yielded the following results (Figure 2): IDC: 24% (95% CI: 18–32%), *I*^2^ = 91.6%; ILC: 22% (95% CI: 13–35%), *I*^2^ = 11.6%; DCIS: 21% (95% CI: 13–32%), *I*^2^ = 25.9%; other histological types: 21% (95% CI: 12–33%), *I*^2^ = 50.5%.

While the overall heterogeneity was high (*I*^2^ = 79.3%, *τ*^2^ = 1.66, *p* < 0.0001), it varied significantly among the subgroups. Notably, ILC demonstrated minimal heterogeneity, indicating consistent findings across studies. In contrast, IDC showed substantial heterogeneity, suggesting considerable methodological or population-based variability between the studies. Given substantial heterogeneity, estimates for IDC should be interpreted as average associations across diverse settings rather than precise effects.

### 3.4 Case–control meta-analysis

**Figure 3:**
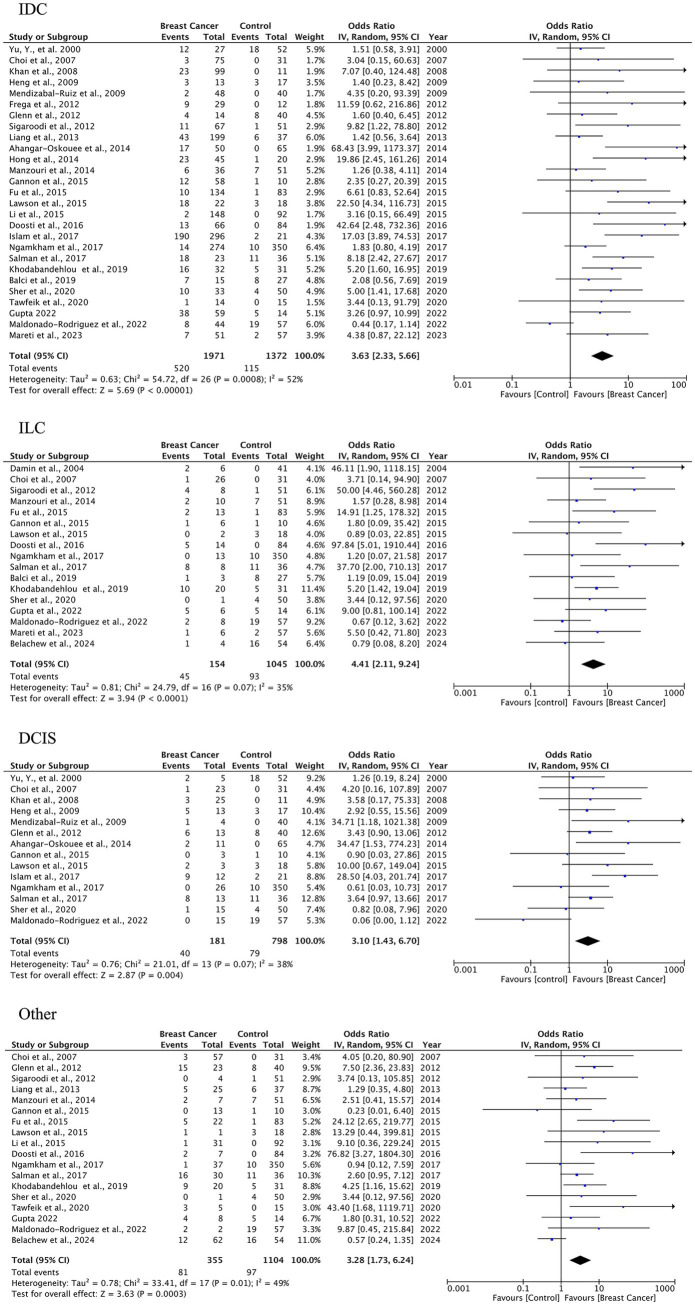
HPV prevalence in breast tissue specimens: case–control analysis by histological subtype (IDC, ILC, DCIS, others).

A total of 27 case–control studies were included in the second analysis phase. Our analytical approach incorporated a three-tiered stratification of case–control studies, evaluating: (1) tumor histology, (2) geographic origin, and (3) HPV genotype (categorized as HPV-16, HPV-18). This multidimensional classification allowed for the detection of type-specific and region-dependent HPV carcinogenesis patterns. The pooled OR for HPV presence in BC tissues compared to control tissues was 3.63 (95% CI: 2.33–5.64, *p* < 0.00001), indicating a significantly higher prevalence of HPV in malignant samples (Figure 3). Subgroup-specific results were as follows: IDC: OR = 3.63 (95% CI: 2.33–5.66), *I*^2^ = 52%; ILC: OR = 4.41 (95% CI: 2.11–9.24), *I*^2^ = 35%; DCIS: OR = 3.10 (95% CI: 1.43–6.70), *I*^2^ = 38%; other types: OR = 3.28 (95% CI: 1.73–6.24), *I*^2^ = 49%. These results indicate a statistically significant association between HPV infection and BC across all histological subtypes. The strongest association was observed in ILC, while DCIS, which is a pre-invasive form, also showed a meaningful link, suggesting the potential role of HPV in early carcinogenic processes.

### 3.5 The incidence of HPV in BC varies by histological type and location

**Figure 4:**
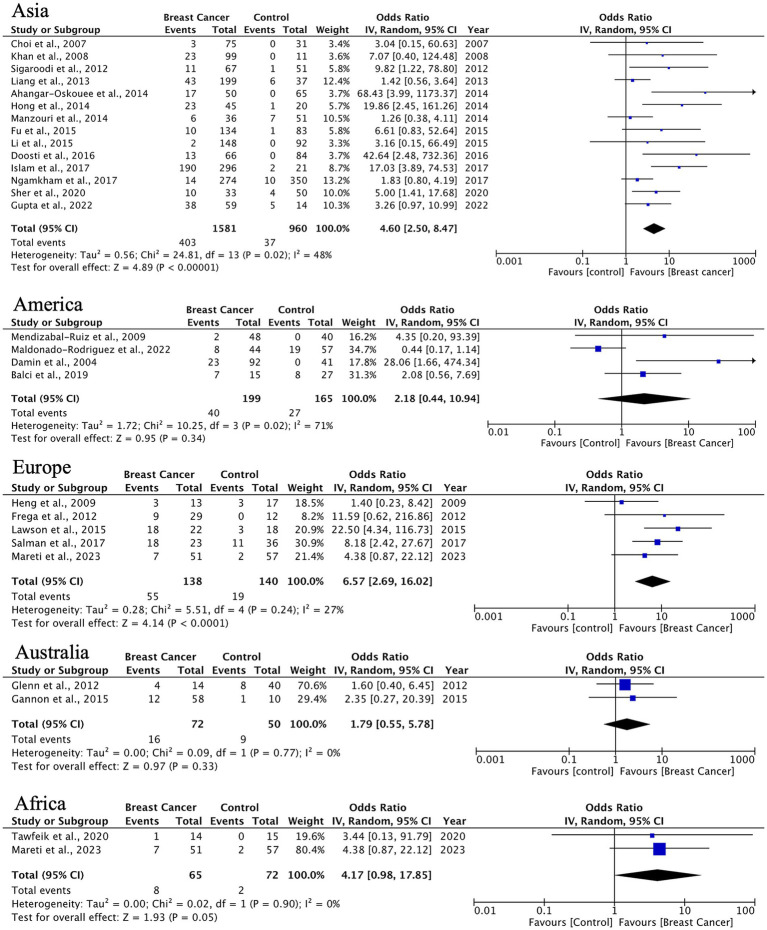
Geographic distribution of human papillomavirus (HPV) prevalence in IDC of the breast. Very wide CIs reflect low event counts and should be interpreted as imprecise estimates rather than robust effects.

In analyzing IDC, the most common BC histotype, we observed an overall HPV prevalence of 24% (95% CI: 18–32%), with the highest detection rates in Asian populations (14 studies, *n* = 1,581 cases) and significant between-study heterogeneity (*I*^2^ = 91.6%). This association was less pronounced in other regions, likely reflecting variations in HPV genotype distribution and detection methodologies (Figure 4).

**Figure 5:**
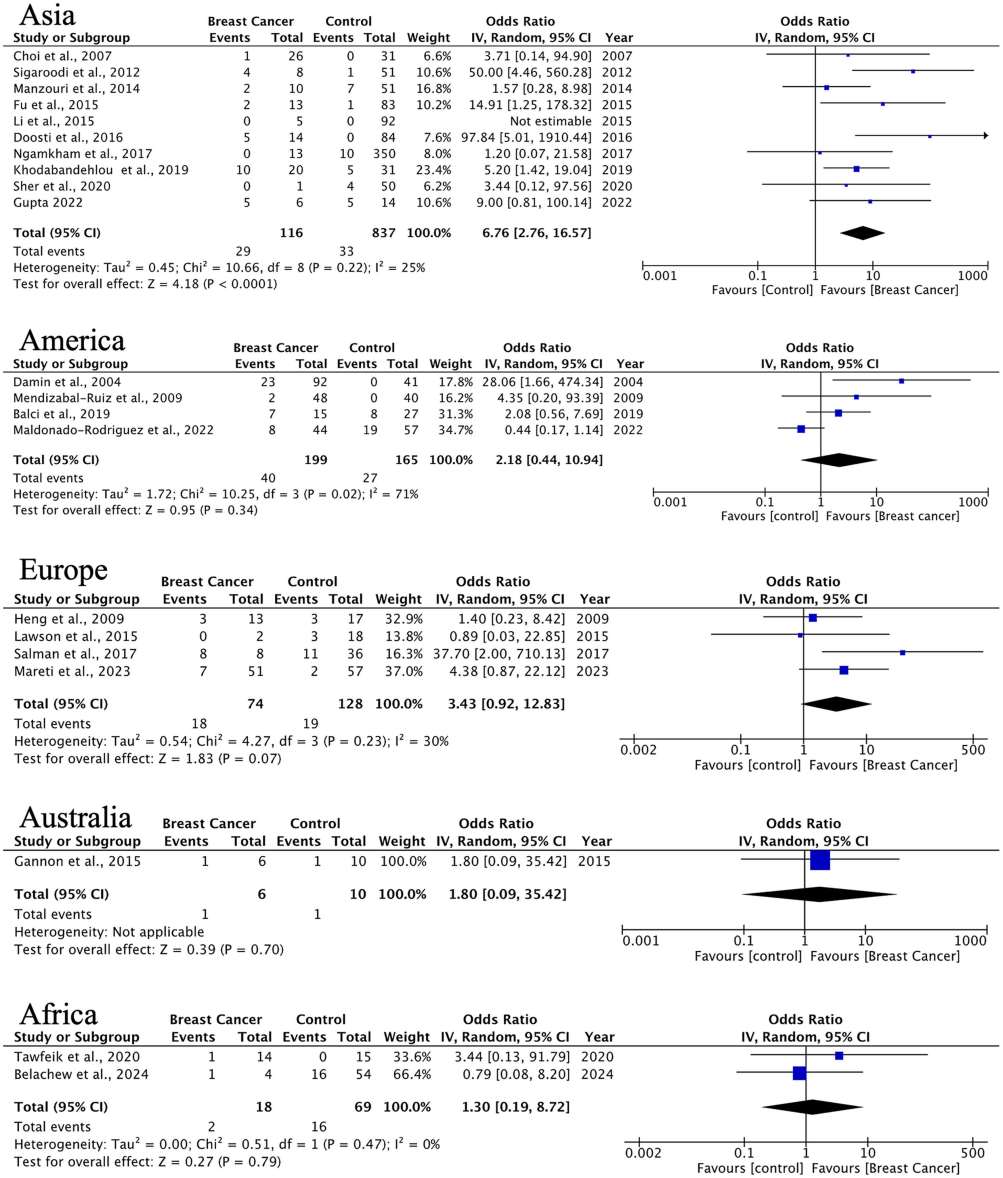
Geographic distribution of human papillomavirus (HPV) prevalence in invasive lobular breast cancer. Very wide CIs reflect low event counts and should be interpreted as imprecise estimates rather than robust effects.

HPV prevalence in ILC exhibits significant geographical variation. Asian studies (10 studies, *n* = 116 cases) demonstrate a strong HPV association (OR = 6.76, 95% CI: 2.76–16.57) with minimal heterogeneity (*I*^2^ = 25%), while other regions present non-significant associations (Americas: OR = 2.18, *p* = 0.33; Europe: OR = 3.43, *p* = 0.07) (Figure 5). The observed trend indicates possible differences in HPV oncogenic mechanisms between ductal and lobular carcinomas.

**Figure 6:**
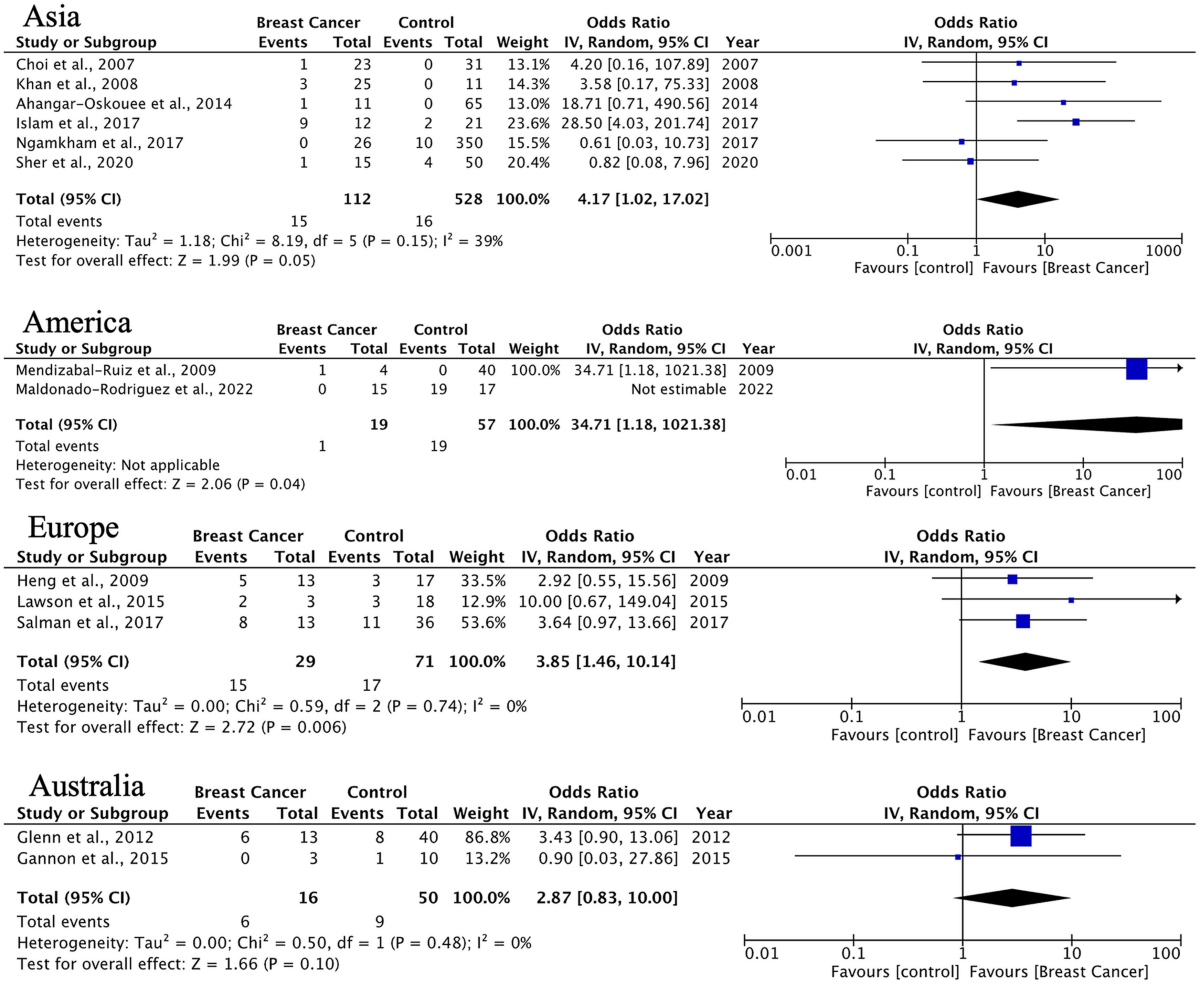
Geographic distribution of human papillomavirus (HPV) prevalence in ductal breast carcinoma in situ. Very wide CIs reflect low event counts and should be interpreted as imprecise estimates rather than robust effects.

For DCIS, we found statistically significant HPV associations in Europe (OR = 3.85, 95% CI: 1.46–10.14, *p* = 0.006) and borderline significance in Asia (OR = 3.29, *p* = 0.05). The European findings demonstrate consistent results across studies (*I*^2^ = 0%), which may support HPV’s potential role in early breast carcinogenesis (Figure 6).

**Figure 7:**
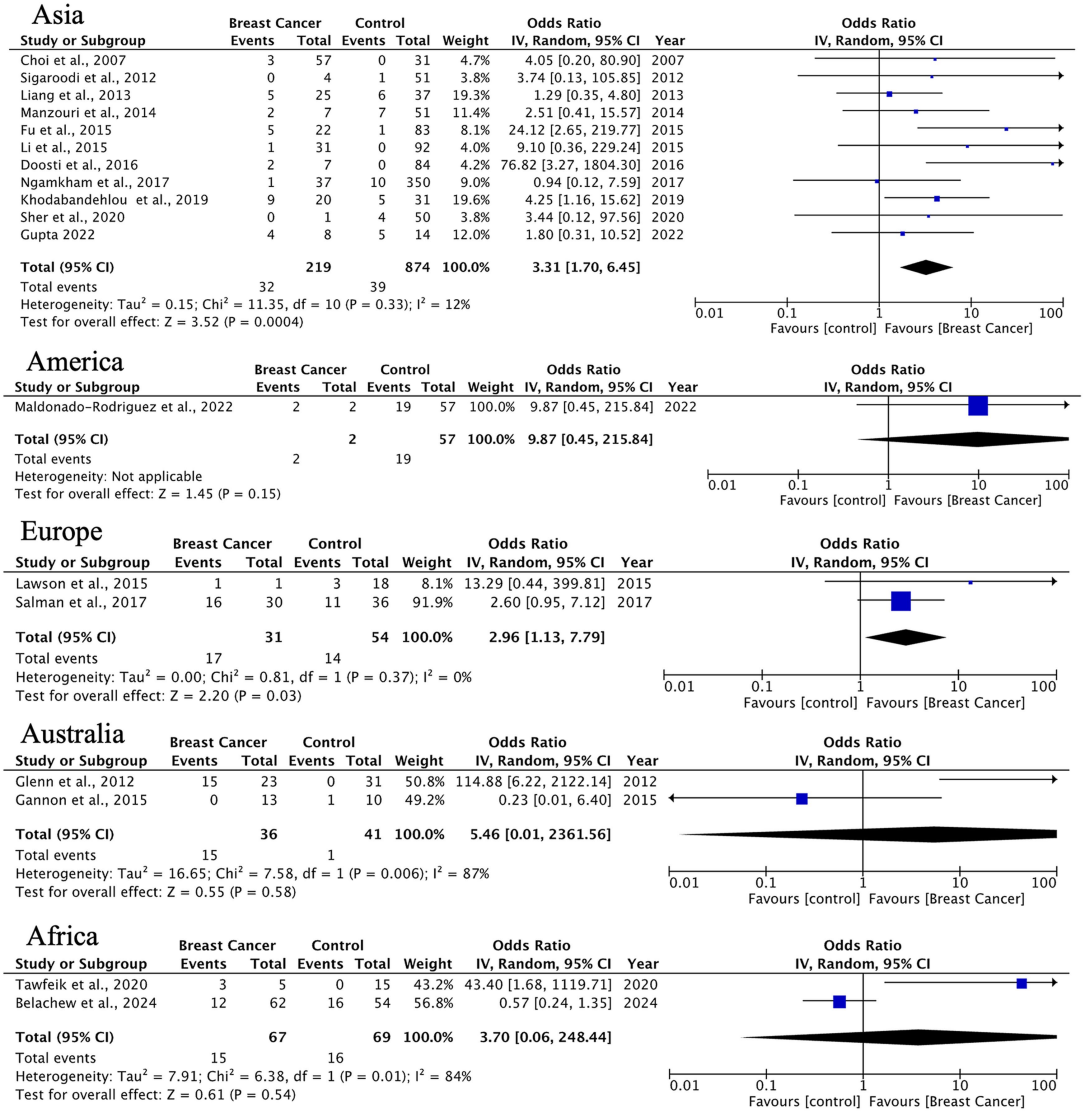
Geographic distribution of human papillomavirus (HPV) prevalence in uncommon breast carcinoma histotypes. Very wide CIs reflect low event counts and should be interpreted as imprecise estimates rather than robust effects.

Other histological types showed significant HPV associations in Asia (OR = 3.31, 95% CI: 1.70–6.45) and Europe (OR = 2.96, 95% CI: 1.13–7.79), although with limited sample sizes. Notably, results from Australia and Africa displayed extreme heterogeneity (*I*^2^ = 84–87%), indicating a need for standardized detection methods and larger studies in these regions (Figure 7).

### 3.6 The global prevalence of HPV 16/18 types in BC and the regional variations observed

**Figure 8:**
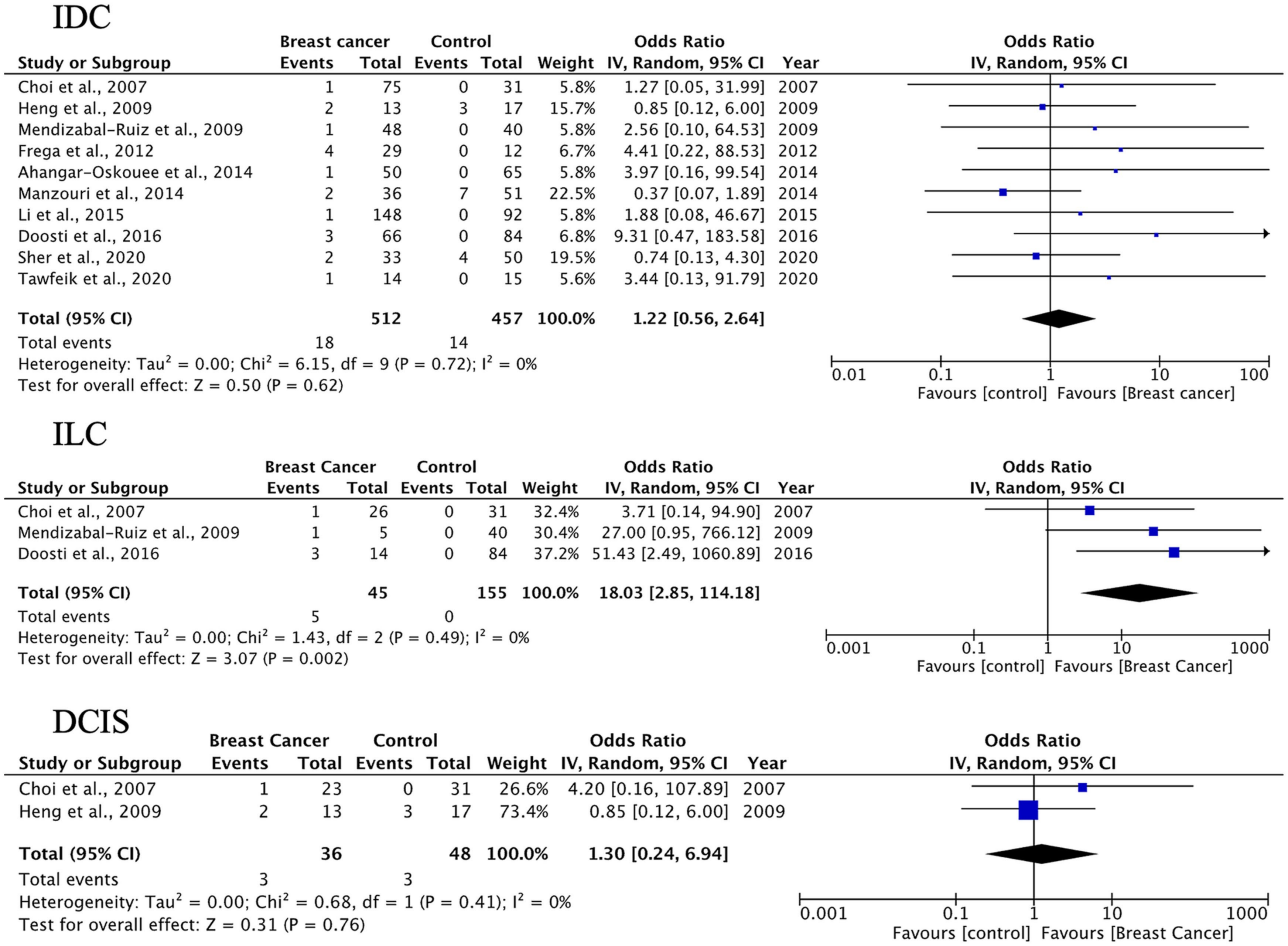
Case–control analysis of human papillomavirus (HPV)-16 frequency in mammary tissue.

The forest plots for HPV-16 and HPV-18 indicate a potential association with BC, though with varying levels of statistical significance and heterogeneity. The data for HPV-16 suggests a less consistent relationship, with the OR reflecting a non-significant positive association (Figure 8). Its clinical heterogeneity reflects diverse histological subtypes and molecular drivers that influence prognosis and therapeutic response.

Transcriptional activity subset. Five studies (~10%) assessed viral transcription (E6/E7 mRNA/protein); three reported positive signals. Where present, associations tended to be stronger, though sample sizes were limited.

**Figure 9:**
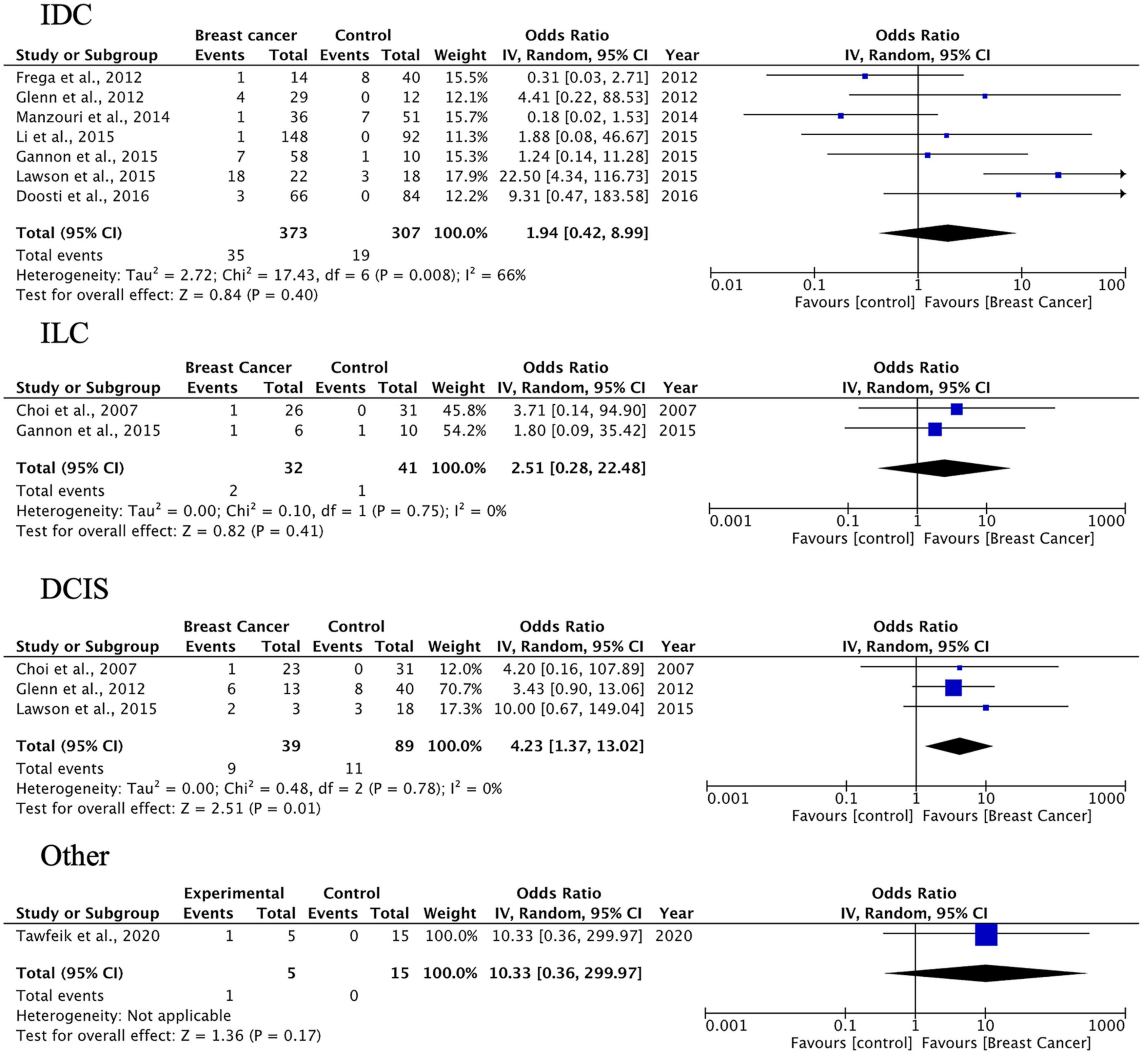
Case–control analysis of human papillomavirus (HPV)-18 frequency in mammary tissue.

HPV-18 appeared more frequently in breast-cancer samples than HPV-16 across several datasets; however, between-study variability precludes definitive conclusions regarding relative strength of association. Further research with larger sample sizes and more uniform study designs would help clarify these associations (Figure 9).

**Figure 10:**
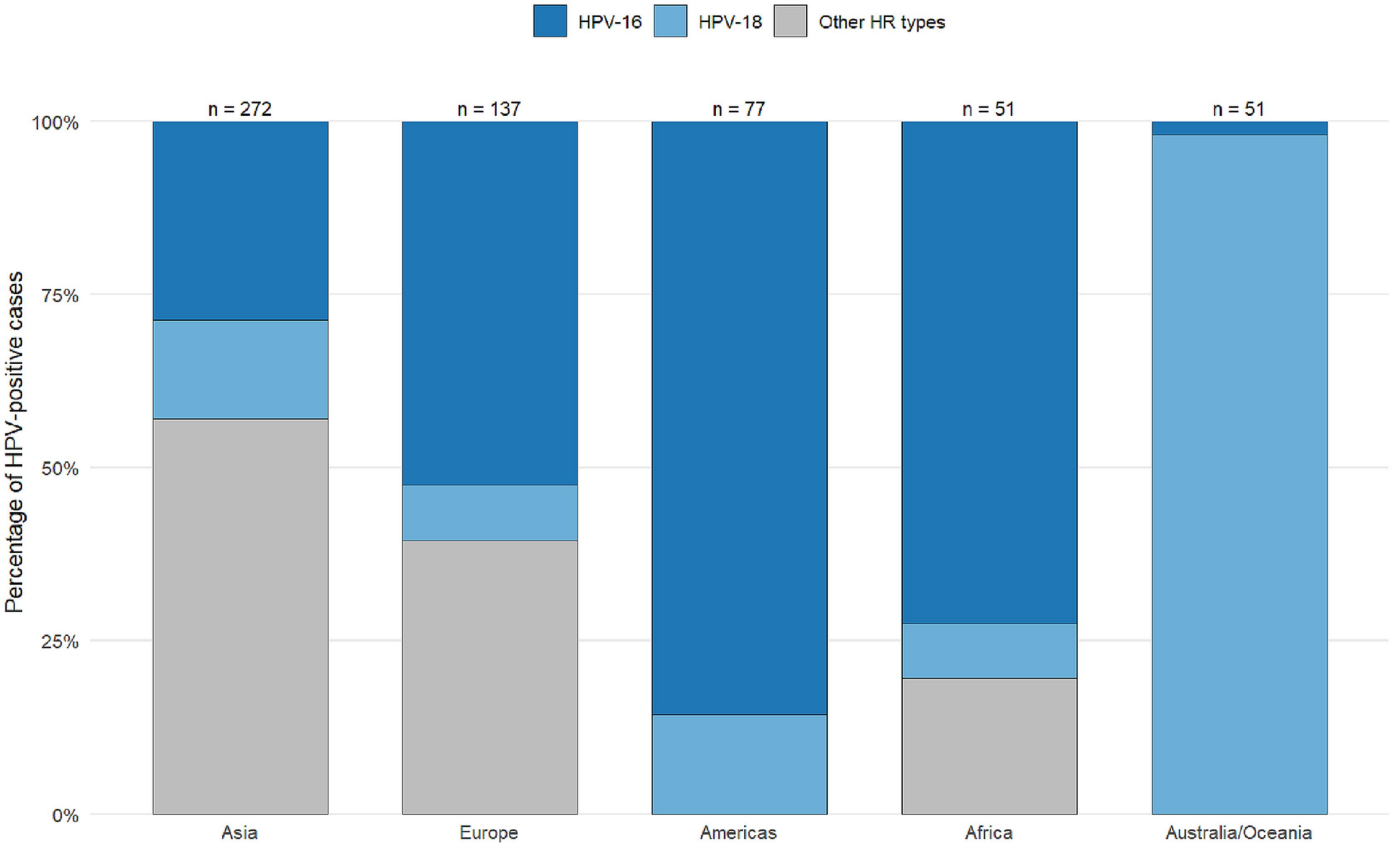
Distribution of human papillomavirus (HPV) types, including those other than 16/18, across various continents worldwide for studies reporting the presence of HPV in breast cancer patients from 1992 to 2022.

Figure 10 illustrate the global distribution of 21 HPV genotypes across five continents: Asia, Africa, the Americas, Europe, and Australia. HPV-16 emerged as the predominant type worldwide, accounting for 254 of 647 total cases (39%), with particularly high prevalence in Asia and Europe. HPV-18 was the second most common type, representing 115 cases (18%). The remaining 19 HPV types showed considerable geographic variation in their distribution patterns, with specific genotypes exhibiting regional predominance. For instance, HPV-31, 33, and 45 demonstrated 3- to 5-fold differences in prevalence between continents, while other oncogenic types (HPV-35, −52, −58) displayed even more pronounced regional clustering. These findings underscore both the universal dominance of HPV-16/18 in breast carcinoma across all studied populations and the distinct regional profiles for less common high-risk HPV types, which may reflect differences in viral evolution, population genetics, or environmental cofactors influencing genotype-specific oncogenesis.

Heterogeneity exploration. In meta-regression of case–control log-ORs, detection method and specimen type were significant sources of variability (omnibus *p* < 0.05). Relative to consensus PCR, nested PCR tended to yield higher effect sizes, while hybrid capture/RT-PCR yielded lower estimates; fresh/frozen tissue showed higher detection than FFPE. Region remained a residual contributor after method/specimen adjustment, indicating both methodological and geographic components to heterogeneity. (Model details in Supplementary Table S4; influence diagnostics and residual plots in Supplementary Figure S1).

### 3.7 Publication bias and sensitivity analyses

**Figure 11:**
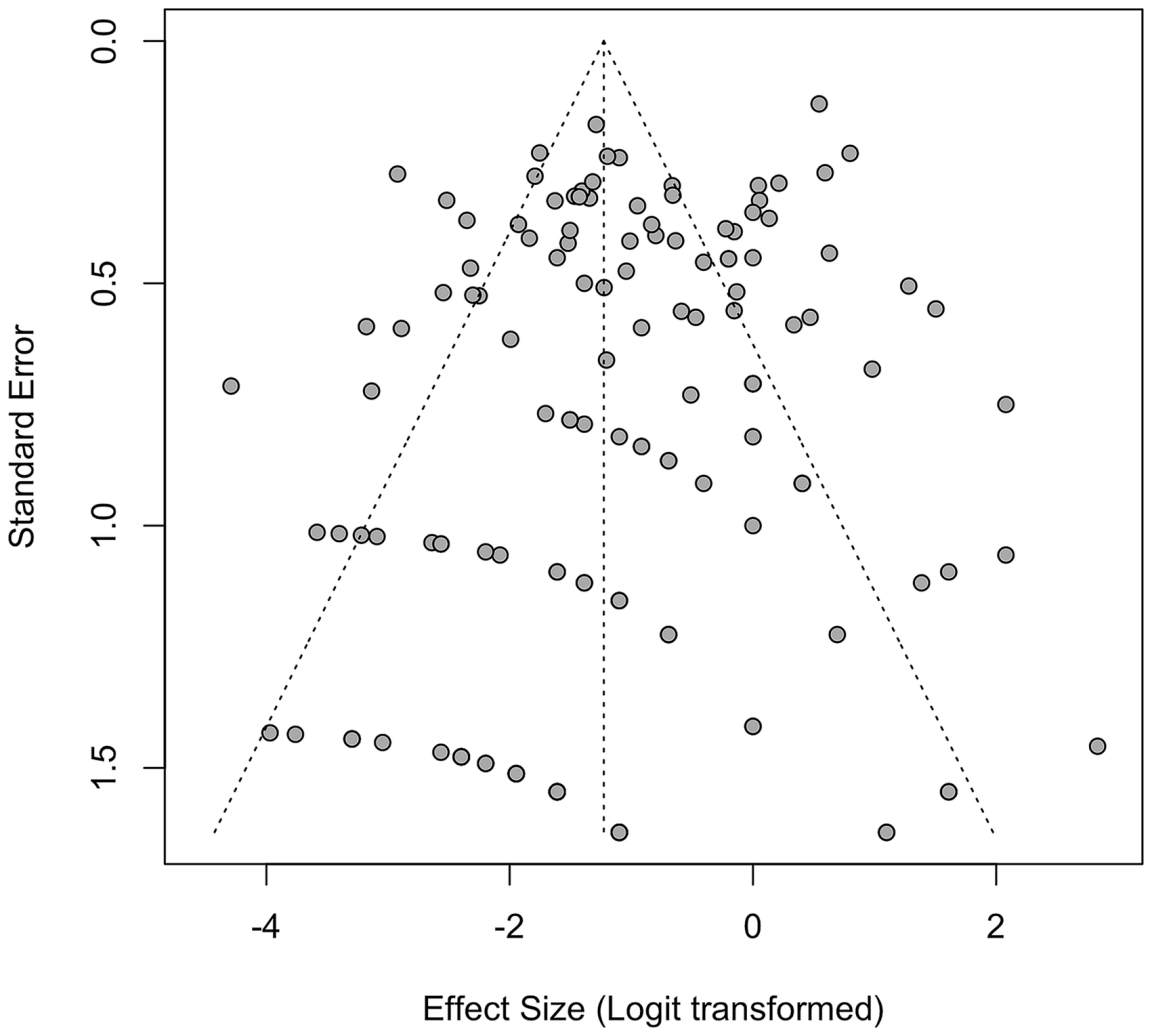
Funnel plot for all studies for studies reporting the presence of human papillomavirus (HPV) in breast cancer patients from 1992 to 2022.

Funnel plot asymmetry was detected for the IDC histotype (Egger’s test: *p* = 0.029; Begg’s test: *p* = 0.425) (Supplementary Table S5 and Figure 11). Trim-and-fill adjustment imputed 10 missing studies, reducing the IDC effect size to 34.9% (26.3–44.5%) while maintaining statistical significance (*τ*^2^ = 2.17, *I*^2^ = 93%). No adjustments were necessary for ILC or DCIS histotypes (Egger’s *p* > 0.3), underscoring the stability of these findings. Publication-bias diagnostics (Egger’s and Begg’s tests, trim-and-fill) were applied to the prevalence model to explore small-study effects; results should be interpreted qualitatively given high heterogeneity (*I*^2^ = 93%).

## 4 Discussion

The pooled prevalence of HPV DNA detected in BC tissues was 23% (95% CI: 18–28%), with the highest rate observed in IDC at 24% (95% CI: 18–32%). HPV positivity was associated with higher odds of BC across histological subtypes, with the strongest signal in ILC. While heterogeneity was substantial for IDC, findings for ILC and DCIS were more consistent, suggesting a potential role for HPV in both invasive lobular disease and *in situ* lesions.

### 4.1 Comparison with prior evidence

Our pooled prevalence aligns with Awan et al. (2023), who reported elevated HPV detection in breast tumors across regions, though direct contrasts are limited by differences in inclusion windows and detection platforms. Importantly, our stratification by histopathology (IDC/ILC/DCIS) and genotype extends the literature by showing: (i) a more stable association in ILC (lower *I*^2^), and (ii) a relatively stronger and more consistent association for HPV-18 than for HPV-16. Findings from Gomes de Oliveira et al. (2022)—who focused on fresh tissues—support that detection can vary by specimen type; our sample-type analyses (see meta-regression below) similarly indicate that fresh/frozen vs. FFPE and detection method contribute materially to heterogeneity. Despite high *I*^2^ for IDC, random-effects pooling is appropriate to summarize between-study variability; consistent directional effects and sensitivity analyses support reporting a pooled estimate, while emphasizing caution.

While our findings corroborate those of Awan et al. (2023), the present review uniquely delineates histology-specific and genotype-specific associations, offering complementary insights into potential subtype-dependent viral oncogenesis. Even after accounting for assay and specimen moderators, the association’s direction remained consistent, suggesting that technical factors alone are unlikely to explain the signal.

### 4.2 Marked geographic variations in HPV prevalence and oncogenic influence

A significant finding of this study is the notable geographical variation in HPV prevalence and its relationship with BC. Studies conducted in Asia showed the highest prevalence and the strongest associations across all histological subtypes. For ILC, the OR reached 6.76 (95% CI: 2.76–16.57) with minimal heterogeneity, indicating a strong link between HPV infection and lobular carcinogenesis in Asian populations. In contrast, European studies primarily linked HPV to DCIS with an OR of 3.85 (95% CI: 1.46–10.14), suggesting a possible role of HPV in the initial phases of tumorigenesis in this group. These regional differences might reflect genuine biological variations in viral affinity, disparities in prevalent HPV genotypes, host genetic susceptibility, or differences in viral detection methodologies. Further exploration of these factors is necessary to clarify the underlying mechanisms driving these regional trends.

The meta-analysis showed distinct patterns regarding the associations of HPV-16 and HPV-18 with BC, emphasizing possible differences in their oncogenic mechanisms. Notably, HPV-18 exhibited a more consistent and stronger association with BC despite considerable variability among studies. This finding may relate to several biological and methodological considerations.

The observed geographic variations in HPV prevalence, particularly the stronger associations in Asian populations, may be influenced by environmental factors exacerbated by global warming, such as increased exposure to air pollutants or UV radiation, which can impair immune responses and potentially enhance HPV persistence (Shirkani and Shirkani, 2024). These environmental carcinogens could act synergistically with viral infections, warranting further investigation into their combined impact on breast carcinogenesis.

### 4.3 Roles of HPV-16 and HPV-18 in breast carcinogenesis

For HPV-18, the stronger association may reflect its unique oncogenic properties in breast tissue. The E6 and E7 oncoproteins of HPV-18 show a high affinity for degrading p53 and inactivating pRb, respectively, which could be particularly efficient in mammary epithelial cells. Additionally, differences in viral genome integration patterns between HPV-18 and other high-risk types may influence its carcinogenic potential. A plausible—but unproven—explanation is differential integration and oncoprotein expression (E6/E7). Only a minority of included studies evaluated transcriptional activity. HPV-18 is known to integrate into host DNA more frequently than HPV-16 in cervical cancer, resulting in sustained expression of E6/E7 and genomic instability (Lagstrom et al., 2021; Bodelon et al., 2016; Liu et al., 2016). A similar mechanism might function in breast tissue, where integrated HPV-18 DNA could drive malignant transformation more effectively. Furthermore, tissue-specific variations in viral entry receptors or host immune responses might favor HPV-18 persistence and oncogenesis in the breast microenvironment (Passmore and Williamson, 2016; Yo and Nuryanto, 2024).

The correlation of HPV-16 with BC was inconsistent and frequently non-significant. This discrepancy may arise from several confounding factors. First, detecting HPV-16 DNA in breast tissue could indicate contamination from adjacent skin or mucosal surfaces rather than involvement in viral carcinogenesis. Given the ubiquity of HPV-16 in anogenital and oropharyngeal cancers, we cannot rule out false-positive results due to sample handling or cross-contamination. Second, methodological differences across studies, such as variations in PCR primers, DNA extraction protocols, or the choice of paraffin-embedded versus fresh tissues, might impact HPV-16 detection rates. For instance, highly sensitive nested PCR studies may overestimate prevalence, whereas studies using sequencing methods could miss low viral loads. Third, biological differences in HPV-16 tropism for breast tissue might limit its oncogenic impact compared to HPV-18. If HPV-16 infects breast cells less efficiently or fails to integrate its genome stably, its contribution to malignant transformation would be weaker.

### 4.4 Clinical and public health implications

Although causality cannot be inferred from detection and case–control designs, HPV-18 appeared more frequently than HPV-16 in breast-cancer samples; however, between-study variability precludes firm conclusions regarding genotype-specific differences. Practically, these data motivate: (i) rigorous, contamination-resistant HPV testing in research biopsies; (ii) careful clinicopathologic correlation; and (iii) HPV vaccination might reduce HPV-related breast lesions if causal links are confirmed; current evidence is insufficient for policy recommendations.\. Until then, over-interpretation for screening or treatment is unwarranted.

### 4.5 Future research

Priorities include: (1) prospective, multi-region studies using standardized pre-analytic handling and orthogonal assays (DNA, E6/E7 mRNA, integration mapping, IHC/RNAscope) to verify active viral oncogenesis; (2) mechanistic models in mammary epithelium (E6/E7 expression, integration, APOBEC footprints); (3) robust case–control matching for sexual, reproductive, and environmental confounders; and (4) individual-participant-data (IPD) meta-analyses to harmonize histology, genotype, and method covariates.

### 4.6 Limitations

Study design & confounding: Predominantly observational designs with limited multivariable control. Differences in DNA extraction, assays (nested/consensus PCR, hybrid capture, RT-PCR), cut-offs, and specimen type (FFPE vs. fresh) influence detection. Our meta-regression indicates these factors materially contribute to heterogeneity. Funnel asymmetry for IDC suggests small-study effects; trim-and-fill attenuated—but did not eliminate—the signal. Over-representation of Asian cohorts may limit generalizability. Presence of viral DNA does not establish causation; markers of transcriptional activity (E6/E7 mRNA), integration, and on-pathway protein changes were inconsistently available. Language restriction to English may have excluded non-English studies (e.g., Chinese, Spanish, Persian) and could affect geographic comparisons. Absence of a formal GRADE assessment means findings are hypothesis-generating and not prescriptive for clinical policy. Early-generation assays may inflate detection in some reports.

## 5 Conclusion

This meta-analysis demonstrates a significant association between human papillomavirus (HPV) infection and breast cancer (BC), with a pooled prevalence of 23% in malignant tissues and a 3.6-fold higher odds of HPV detection compared with non-cancerous controls. These findings support a potential association of HPV in BC and underscore the need to strengthen the causality-focused evidence base through standardized detection protocols and mechanistic studies. Future research should prioritize large multi-regional cohorts and experimental models to determine whether HPV acts as a causal agent or co-factor in BC pathogenesis.

## Data Availability

The original contributions presented in the study are included in the article/Supplementary material, further inquiries can be directed to the corresponding authors.
