# Supplementary material for: The oncogenic role of human papillomavirus in breast cancer: a comprehensive systematic review and meta-analysis
Source: Front Microbiol. 2025 Nov 14;16:1712118. doi: 10.3389/fmicb.2025.1712118 (PMC12660267; doi:10.3389/fmicb.2025.1712118)
Supplement: Supplementary file 1 [file Table_1.docx]

Table S1. Distribution of human papillomavirus (HPV) genotypes and co-infections reported across studies reporting the presence of HPV in breast cancer patients from 1992 to 2022

| Authors, (reference) | HPV-16 | HPV-18 | HPV-33 | HPV-31 | HPV-35 | HPV-39 | HPV-66 | HPV-70 | HPV-50 | HPV-45 | HPV-51 | HPV-56 | HPV-6 | HPV-23 | HPV-4 | HPV-26 | HPV-11 | HPV-15 | HPV-124 | HPV-24 | HPV-5 | HPV-59 | HPV-58 | HPV-38 | HPV-22 | HPV-52 | Co-Infection | Total Cases |
| --- | --- | --- | --- | --- | --- | --- | --- | --- | --- | --- | --- | --- | --- | --- | --- | --- | --- | --- | --- | --- | --- | --- | --- | --- | --- | --- | --- | --- |
| Aguayo et al. (1) | 4 | - | - | - | - | - | - | - | - | - | - | - | - | - | - | - | - | - | - | - | - | - | - | - | - | - | - | 4 |
| Ahangar-Oskouee et al. (2) | 1 | - | - | - | 1 | - | - | - | - | - | - | - | 17 | - | - | - | 1 | - | - | - | - | - | - | - | - | 1 | 1 | 22 |
| Akil et al. (3) | 9 | 11 | 8 | 58 | 39 | - | - | - | - | - | - | - | - | - | - | - | - | - | - | - | - | - | - | - | - | - | - | 69 |
| Alinezhadi et al. (4) | 2 |  | 3 | 2 |  |  |  |  |  |  |  |  |  |  |  |  | 1 |  |  |  |  |  |  |  |  |  |  | 63 |
| Antonsson et al. (5) | - | 18 | - | - | - | - | - | - | - | - | - | - | - | - | - | - | - | - | - | - | - | - | - | - | - | - | - | 27 |
| Balci et al. (6) | - | - | - | - | - | - | - | - | - | - | - | - | - | - | - | 5 | - | - | - | - | - | - | - | - | - | - | 3 | 8 |
| Baltzell et al. (7) | 6 | - | - | - | - | - | - | - | - | - | - | - | - | - | - | - | - | - | - | - | - | - | - | - | - | - | - | 6 |
| Belachew et al. (8) | 12 |  |  |  |  |  |  |  |  |  |  |  |  |  |  |  |  |  |  |  |  | 1 |  |  |  |  |  | 14 |
| Choi et al. (9) | 1 | 2 | - | 1 | - | - | - | 2 | - | - | - | - | - | - | - | - | - | - | - | - | - | 1 | - | - | - | - | 1 | 8 |
| Damin et al. (10) | 14 | 10 | - | - | - | - | - | - | - | - | - | - | - | - | - | - | - | - | - | - | - | - | - | - | - | - | 1 | 25 |
| de Villiers et al.(11) | 17 | - | - | - | - | - | - | - | - | - | - | - | - | - | - | - | - | - | - | - | - | - | - | - | - | - | 8 | 25 |
| Doosti et al. (12) | 7 | 3 | - | - | - | - | - | - | - | - | - | - | 9 | - | - | - | 1 | - | - | - | - | - | - | - | - | - | - | 20 |
| Duò et al. (13) | - | - | - | - | - | - | 2 | - | - | - | - | - | - | - | - | - | - | - | - | - | - | - | - | - | - | - | - | 2 |
| Elagali et al. (14) | 5 | 3 | - | - | - | - | - | - | - | - | - | - | - | - | - | - | 1 | - | - | - | - | - | 4 | - | - | - | - | 13 |
| Fernandes et al. (15) | - | - | - | - | - | - | - | - | - | - | - | - | - | - | - | - | - | - | - | - | - | - | - | - | - | - | 10 | 10 |
| Frega et al. (16) | 3 | 1 | - | - | - | - | - | - | - | - | 1 | 1 | 1 | - | - | - | - | - | - | - | - | - | - | - | - | - | 2 | 9 |
| Fu et al. (17) | - | - | - | - | - | - | - | - | - | - | - | - | - | - | - | - | - | - | - | - | - | - | 17 | - | - | - | 8 | 25 |
| Gannon et al.(18) | - | - | - | - | - | - | - | - | - | - | - | - | - | - | - | - | - | - | - | - | - | - | - | - | - | - | 13 | 13 |
| Ghaffari et al. (19) | - | - | - | - | - | - | - | - | - | - | - | - | - | - | - | - | - | - | - | - | - | - | - | - | - | - | 4 | 4 |
| Glenn et al. (20) | - | 25 | - | - | - | - | - | - | - | - | - | - | - | - | - | - | - | - | - | - | - | - | - | - | - | - | - | 25 |
| Gupta et al. (21) |  |  |  |  |  |  |  |  |  |  |  |  |  |  |  |  |  |  |  |  |  |  |  |  |  |  |  | 48 |
| Habyarimana et al. (22) | 17 | - | 3 | 2 | - | - | - | - | - | - | - | - | - | - | - | - | - | - | - | - | - | - | - | - | - | - | - | 22 |
| Heng et al. (23) | 1 | 7 | - | - | - | - | - | - | - | - | - | - | - | - | - | - | - | - | - | - | - | - | - | - | - | - | - | 8 |
| Hennig et al. (24) | 19 | - | - | - | - | - | - | - | - | - | - | - | - | - | - | - | - | - | - | - | - | - | - | - | - | - | - | 19 |
| Herrera-Goepfert et al. (25) | 15 | - | - | - | - | - | - | - | - | - | - | - | - | - | - | - | - | - | - | - | - | - | - | - | - | - | 2 | 17 |
| Herrera-Goepfert et al. (26) | 7 | 1 | - | - | - | - | - | - | - | - | - | - | - | - | - | - | - | - | - | - | - | - | - | - | - | - | - | 8 |
| Hong and Tang (27) | - | - | - | - | - | - | - | - | - | - | - | - | - | - | - | - | - | - | - | - | - | - | - | - | - | - | 23 | 23 |
| Islam et al. (28) | - | - | - | - | - | - | - | - | - | - | - | - | - | - | - | - | - | - | - | - | - | - | - | - | - | - | 203 | 203 |
| Khan et al. (29) | 13 | 1 | - | - | - | - | - | - | - | - | - | - | 1 | - | - | - | - | - | - | 11 | - | - | - | - | - | - | - | 26 |
| Khodabandehlou et al. (30) | 13 | 16 | 4 | - | - | - | - | - | - | - | - | - | - | - | - | - | - | - | - | - | - | - | - | - | - | - | 2 | 35 |
| Kroupis et al. (31) | 14 | - | - | - | - | - | - | - | - | - | - | - | - | - | - | - | - | - | - | - | - | - | - | - | - | - | 3 | 17 |
| Lawson et al. (32) | - | - | - | - | - | - | - | - | - | - | - | - | - | - | - | - | - | - | - | - | - | - | - | - | - | - | 13 | 13 |
| Li et al. (33) | 2 | 1 | - | - | - | - | - | - | - | - | - | - | - | - | - | - | - | - | - | - | - | - | - | - | - | - | - | 3 |
| Liang et al. (34) | - | - | - | - | - | - | - | - | - | - | - | - | - | - | - | - | - | - | - | - | - | - | - | - | - | - | 48 | 48 |
| Maldonado-Rodriguez et al. (35) |  |  |  |  |  |  |  |  |  |  |  |  |  |  |  |  |  |  |  |  |  |  |  |  |  |  | 5 | 12 |
| Manzouri et al. (36) | 2 | 1 | 1 | - | 1 | - | - | - | - | - | - | - | - | - | - | - | 2 | - | - | - | - | - | - | - | - | - | 6 | 10 |
| Mareti et al. (37) | 5 | 2 |  |  |  |  |  |  |  |  |  |  |  |  |  |  |  |  |  |  |  |  |  |  |  |  |  | 7 |
| Mendizabal-Ruiz et al. (38) | - | - | - | - | - | - | - | - | - | - | - | - | - | - | - | - | - | - | - | - | - | - | - | - | - | - | 3 | 3 |
| Nascimento et al. (39) | 20 |  |  |  |  |  |  |  |  |  |  |  |  |  |  |  |  |  |  |  |  |  |  |  |  |  |  | 20 |
| Naushad et al. (40) | - | - | - | - | - | - | - | - | - | - | - | - | - | - | - | - | - | - | - | - | - | - | - | - | - | - | 45 | 45 |
| Ngamkham et al. (41) | - | - | - | - | - | - | - | - | - | - | - | - | - | - | - | - | - | - | - | - | - | - | - | - | - | - | 15 | 15 |
| Pereira Suarez et al. (42) | - | - | - | - | - | - | - | - | - | - | - | - | - | - | - | - | - | - | - | - | - | - | - | - | - | - | 16 | 16 |
| Salman et al. (43) | 7 | 8 | 3 | 5 | 7 | 13 | - | - | - | 8 | - | 1 | - | - | - | - | - | - | - | - | - | 7 | 4 | - | - | 2 | - | 35 |
| Sher et al. (44) | - | - | - | - | - | - | - | - | - | - | - | - | - | - | - | - | - | - | - | - | - | - | - | - | - | - | 10 | 10 |
| Sigaroodi et al. (45) | 4 | 4 | - | - | - | - | - | - | - | - | - | - | 2 | 2 | - | - | 1 | 1 | 1 | - | - | - | - | - | - | - | - | 15 |
| Tawfeik et al. (46) | 3 | 1 | - | - | - | - | - | - | - | - | - | - | - | - | - | - | - | - | - | - | - | - | - | - | - | - | - | 4 |
| Wang et al. (47) | - | - | - | - | - | - | - | - | - | - | - | - | - | - | - | - | - | - | - | - | - | - | - | - | - | - | 14 | 14 |
| Widschwendter et al. (48) | 7 | - | - | - | - | - | - | - | - | - | - | - | - | - | - | - | - | - | - | - | - | - | - | - | - | - | - | 7 |
| Yu et al. (49) | - | - | 18 | - | - | - | - | - | - | - | - | - | - | - | - | - | - | - | - | - | - | - | - | - | - | - | - | 18 |

Table S2. Quality assessment of cross-sectional studies utilizing the JBI critical appraisal checklist for studies reporting the presence of HPV in breast cancer patients from 1992 to 2022

| Authors, year (reference) | Were the criteria for inclusion in the sample clearly defined? | Were the study subjects and the setting described in detail? | Was the exposure measured in a valid and reliable way? | Were objective, standard criteria used for measurement of the condition? | Were confounding factors identified? | Were strategies to deal with confounding factors stated? | Were outcomes measured in a valid and reliable way? | Was appropriate statistical analysis used? | Total |
| --- | --- | --- | --- | --- | --- | --- | --- | --- | --- |
| Aguayo et al., 2011 (1) | 1 | 1 | 1 | 1 | 0.5 | 0 | 1 | 0.5 | 6/8 |
| Akil et al., 2008 (3) | 1 | 1 | 1 | 1 | 0.5 | 0 | 1 | 0.5 | 6/8 |
| Alinezhadi et al., 2022 (4) | 1 | 1 | 1 | 1 | 0.5 | 0 | 1 | 0.5 | 6/8 |
| Antonsson et al., 2011 (5) | 1 | 1 | 1 | 1 | 0.5 | 0 | 1 | 0.5 | 6/8 |
| Baltzell et al., 2012 (7) | 1 | 1 | 1 | 1 | 0.5 | 0 | 1 | 0.5 | 6/8 |
| de Villiers et al., 2005 (11) | 1 | 1 | 1 | 1 | 0.5 | 0 | 1 | 0.5 | 6/8 |
| Duo et al., 2008 (13) | 1 | 1 | 1 | 1 | 0.5 | 0 | 1 | 0.5 | 6/8 |
| Elagali et al., 2021 (14) | 1 | 1 | 1 | 1 | 0.5 | 0 | 1 | 0.5 | 6/8 |
| Fernandes et al., 2015 (15) | 1 | 1 | 1 | 1 | 0.5 | 0 | 1 | 0.5 | 6/8 |
| Fu et al., 2015 (17) | 1 | 1 | 1 | 1 | 0.5 | 0 | 1 | 0.5 | 6/8 |
| Ghaffari et al., 2018 (19) | 1 | 1 | 1 | 1 | 0.5 | 0 | 1 | 0.5 | 6/8 |
| Habyarimana et al., 2018 (22) | 1 | 1 | 1 | 1 | 0.5 | 0 | 1 | 0.5 | 6/8 |
| Hennig et al., 1999 (24) | 1 | 1 | 1 | 1 | 0.5 | 0 | 1 | 0.5 | 6/8 |
| Herrera-Goepfert et al., 2011 (25) | 1 | 1 | 1 | 1 | 0.5 | 0 | 1 | 0.5 | 6/8 |
| Herrera-Goepfert et al., 2013 (26) | 1 | 1 | 1 | 1 | 0.5 | 0 | 1 | 0.5 | 6/8 |
| Kroupis et al., 2006 (31) | 1 | 1 | 1 | 1 | 0.5 | 0 | 1 | 0.5 | 6/8 |
| Nascimento et al., 2024 (39) | 1 | 1 | 1 | 1 | 0.5 | 0 | 1 | 0.5 | 6/8 |
| Ngamkham et al., 2017 (41) | 1 | 1 | 1 | 1 | 0.5 | 0 | 1 | 0.5 | 6/8 |
| Pereira Suarez et al., 2013 (42) | 1 | 1 | 1 | 1 | 0.5 | 0 | 1 | 0.5 | 6/8 |
| Wang et al., 2017 (47) | 1 | 1 | 1 | 1 | 0.5 | 0 | 1 | 0.5 | 6/8 |
| Widschwendter et al., 2004 (48) | 1 | 1 | 1 | 1 | 0.5 | 0 | 1 | 0.5 | 6/8 |
| Yu et al., 2000 (49) | 1 | 1 | 1 | 1 | 0.5 | 0 | 1 | 0.5 | 6/8 |

Interpretation: 7–8 points: High quality; 5–6.5 points: Moderate quality; <5 points: Low quality

Table S3. Quality assessment of included case-control studies utilizing the Newcastle-Ottawa Scale (NOS) for studies reporting the presence of HPV in breast cancer patients from 1992 to 2022

| Authors, year (reference) | Selection | | | | Comparability | | Exposure | | | Total |
| --- | --- | --- | --- | --- | --- | --- | --- | --- | --- | --- |
|  | Case definition adequate | Representativeness of the cases | Selection of controls | Definition of controls | Comparability of cases and controls | Control for additional confounders | Ascertainment of exposure | Same method of ascertainment for groups | Non-response rate |  |
| Ahangar-Oskouee et al., 2014 (2) | Yes | Yes | Yes | Yes | No | No | Yes | Yes | No | 6/9 |
| Balci et al., 2019 (6) | Yes | Yes | Yes | Yes | No | No | Yes | Yes | No | 6/9 |
| Belachew et al., 2024 (8) | Yes | Yes | Yes | Yes | No | No | Yes | Yes | No | 6/9 |
| Choi et al., 2007 (9) | Yes | Yes | Yes | No | No | No | Yes | Yes | No | 5/9 |
| Damin et al., 2004 (10) | Yes | Yes | Yes | Yes | No | No | Yes | Yes | No | 6/9 |
| Doosti et al., 2016 (12) | Yes | Yes | Yes | Yes | No | No | Yes | Yes | No | 6/9 |
| Frega et al., 2012 (16) | Yes | Yes | Yes | Yes | No | No | Yes | Yes | No | 6/9 |
| Gannon et al., 2015 (18) | Yes | Yes | Yes | Yes | No | No | Yes | Yes | No | 6/9 |
| Glenn et al., 2012 (20) | Yes | Yes | Yes | Yes | No | No | Yes | Yes | No | 6/9 |
| Gupta et al., 2021 (21) | Yes | Yes | No | No | No | No | Yes | No | No | 3/9 |
| Hennig et al., 1999 (24) | Yes | Yes | Yes | Yes | No | No | Yes | Yes | No | 6/9 |
| Hong and Tang, 2014 (27) | Yes | Yes | Yes | Yes | No | No | Yes | Yes | No | 6/9 |
| Islam et al., 2017 (28) | Yes | Yes | Yes | Yes | No | No | Yes | Yes | No | 6/9 |
| Khan et al., 2008 (29) | Yes | Yes | No | Yes | No | No | Yes | Yes | No | 5/9 |
| Khodabandehlou et al., 2019 (30) | Yes | Yes | Yes | Yes | Yes | No | Yes | Yes | No | 7/9 |
| Lawson et al., 2015 (32) | Yes | Yes | Yes | Yes | Yes | No | Yes | Yes | No | 7/9 |
| Li et al., 2015 (50) | Yes | Yes | Yes | Yes | No | No | Yes | Yes | No | 6/9 |
| Liang et al., 2013 (34) | Yes | Yes | Yes | Yes | No | No | Yes | Yes | No | 6/9 |
| Maldonado-Rodriguez et al., 2022 (35) | Yes | Yes | Yes | Yes | No | No | Yes | Yes | No | 6/9 |
| Manzouri et al., 2014 (36) | Yes | Yes | Yes | Yes | No | No | Yes | Yes | No | 6/9 |
| Mareti et al., 2023 (37) | Yes | Yes | Yes | Yes | No | No | Yes | Yes | No | 6/9 |
| Mendizabal-Ruiz et al., 2009 (38) | Yes | Yes | Yes | Yes | No | No | Yes | Yes | No | 6/9 |
| Naushad et al., 2017 (40) | Yes | Yes | Yes | Yes | No | No | Yes | Yes | No | 6/9 |
| Salman et al., 2017 (43) | Yes | Yes | Yes | Yes | No | No | Yes | Yes | No | 6/9 |
| Sher et al., 2020 (44) | Yes | Yes | Yes | Yes | No | No | Yes | Yes | No | 6/9 |
| Sigaroodi et al., 2012 (45) | Yes | Yes | Yes | Yes | No | No | Yes | Yes | No | 6/9 |
| Tawfeik et al., 2020 (46) | Yes | Yes | Yes | Yes | No | No | Yes | Yes | No | 6/9 |

Table S4. Meta-regression exploring sources of heterogeneity in the association between HPV infection and breast cancer across included case–control studies

| Moderator variable | Category (reference) | Regression coefficient (β) | 95 % Confidence Interval | p-value | Interpretation |
| --- | --- | --- | --- | --- | --- |
| Detection method | Consensus PCR (reference) | — | — | — | — |
|  | Nested PCR | +0.48 | 0.09 to 0.87 | 0.017 | Higher odds ratios; nested PCR yields greater HPV detection. |
|  | Hybrid capture / RT-PCR | −0.62 | −1.12 to −0.12 | 0.014 | Lower odds ratios; less sensitive than consensus PCR. |
|  | Sequencing / In situ hybridization | −0.35 | −0.91 to 0.21 | 0.214 | Non-significant difference. |
| Specimen type | FFPE (reference) | — | — | — | — |
|  | Fresh / frozen | +0.57 | 0.14 to 1.00 | 0.009 | Higher detection in fresh/frozen tissues. |
| Geographic region | Asia (reference) | — | — | — | — |
|  | Europe | −0.29 | −0.78 to 0.19 | 0.241 | Slightly lower but non-significant effect. |
|  | Americas | −0.36 | −0.82 to 0.10 | 0.128 | Trend toward smaller association. |
|  | Africa / Australia | −0.18 | −0.72 to 0.36 | 0.507 | No significant difference. |
| Model statistics | — | — | — | — | — |
|  | Between-study variance (τ²) | 0.42 | — | — | Residual heterogeneity reduced by 38 % vs base model. |
|  | Omnibus test for moderators (QM) | 19.4 (df = 7) | — | — | p = 0.006 |
|  | Residual I² | 61 % | — | — | — |

Abbreviations: HPV = human papillomavirus; FFPE = formalin-fixed paraffin-embedded; PCR = polymerase chain reaction; RT-PCR = reverse-transcription PCR.

Model specification: random-effects meta-regression (REML estimator) of log(OR) on moderators = detection method + specimen type + geographic region (k = 27).

Table S5. Assessment of publication bias results across histological groups for studies reporting the presence of HPV in breast cancer patients from 1992 to 2022

| Group | Egger's Test (p-value) | Begg's Test (p-value) | Heterogeneity (τ²) | Conclusion |
| --- | --- | --- | --- | --- |
| IDC | 0.029* (Bias = -2.67) | 0.425 | 10.96 (high) | Significant bias detected |
| ILC | 0.335 | 0.618 | 1.69 (moderate) | No significant bias |
| DCIS | 0.343 | 0.738 | 1.75 (moderate) | No significant bias |
| Other | 0.304 | 0.964 | 2.51 (moderate) | No significant bias |

Abbreviations: IDC: Invasive Ductal Carcinoma; ILC: Invasive Lobular Carcinoma; DCIS: Ductal Carcinoma In Situ; τ²: Tau-squared (measure of between-study variance)





Figure S1. Bubble plot of meta-regression showing the influence of detection method and specimen type on HPV–breast-cancer association. Meta-regression bubble plot illustrating the effects of detection method and specimen type on the log-odds ratio (log OR) of HPV detection in breast cancer case–control studies. Each circle represents one study (n = 27). Bubble size is proportional to study weight (inverse of the within-study variance). Panels show adjusted log ORs after random-effects modeling (REML). Nested PCR and fresh/frozen specimen studies cluster toward higher log ORs, indicating stronger observed associations, whereas hybrid-capture and RT-PCR studies cluster near zero. Regression line (solid) depicts the fitted moderator effect; 95 % confidence band (shaded) demonstrates the uncertainty range. Residual heterogeneity (I² = 61 %) remains after accounting for moderators, but between-study variance (τ²) is reduced by ≈ 38 % compared with the base model. Abbreviations: FFPE = formalin-fixed paraffin-embedded; PCR = polymerase chain reaction; RT-PCR = reverse-transcription PCR; OR = odds ratio.

**References**

1. Aguayo F, Khan N, Koriyama C, Gonzalez C, Ampuero S, Padilla O, Solis L, Eizuru Y, Corvalan A, Akiba S. Human papillomavirus and Epstein-Barr virus infections in breast cancer from chile. Infect Agent Cancer. 2011;6(1):7. doi:10.1186/1750-9378-6-7

2. Ahangar-Oskouee M, Shahmahmoodi S, Jalilvand S, Mahmoodi M, Ziaee AA, Esmaeili HA, Keshtvarz M, Pishraft-Sabet L, Yousefi M, Mollaei-Kandelous Y, Mokhtari-Azad T, Nategh R. No detection of 'high-risk' human papillomaviruses in a group of Iranian women with breast cancer. Asian Pac J Cancer Prev. 2014;15(9):4061-5. doi:10.7314/apjcp.2014.15.9.4061

3. Akil N, Yasmeen A, Kassab A, Ghabreau L, Darnel AD, Al Moustafa AE. High-risk human papillomavirus infections in breast cancer in Syrian women and their association with Id-1 expression: a tissue microarray study. Br J Cancer. 2008;99(3):404-7. doi:10.1038/sj.bjc.6604503

4. Alinezhadi M, Makvandi M, Kaydani GA, Jazayeri SN, Charostad J, Talaeizadeh AT, Angali KA. Detection of High-Risk Human Papillomavirus DNA in Invasive Ductal Carcinoma Specimens. Asian Pac J Cancer Prev. 2022;23(9):3201-7. doi:10.31557/APJCP.2022.23.9.3201

5. Antonsson A, Spurr TP, Chen AC, Francis GD, McMillan NA, Saunders NA, Law M, Bennett IC. High prevalence of human papillomaviruses in fresh frozen breast cancer samples. J Med Virol. 2011;83(12):2157-63. doi:10.1002/jmv.22223

6. Balci FL, Uras C, Feldman SM. Is human papillomavirus associated with breast cancer or papilloma presenting with pathologic nipple discharge? Cancer Treat Res Commun. 2019;19:100122. doi:10.1016/j.ctarc.2019.100122

7. Baltzell K, Buehring GC, Krishnamurthy S, Kuerer H, Shen HM, Sison JD. Limited evidence of human papillomavirus in [corrected] breast tissue using molecular in situ methods. Cancer. 2012;118(5):1212-20. doi:10.1002/cncr.26389

8. Belachew EB, Desta AF, Mulu A, Deneke DB, Tefera DA, Alemu A, Anberber E, Beshah D, Girma S, Sewasew DT, Tessema TS, Howe R. High rate of high-risk human papillomavirus among benign and breast cancer patients in Ethiopia. PLoS One. 2024;19(3):e0298583. doi:10.1371/journal.pone.0298583

9. Choi YL, Cho EY, Kim JH, Nam SJ, Oh YL, Song SY, Yang JH, Kim DS. Detection of human papillomavirus DNA by DNA chip in breast carcinomas of Korean women. Tumour Biol. 2007;28(6):327-32. doi:10.1159/000124238

10. Damin AP, Karam R, Zettler CG, Caleffi M, Alexandre CO. Evidence for an association of human papillomavirus and breast carcinomas. Breast Cancer Res Treat. 2004;84(2):131-7. doi:10.1023/B:BREA.0000018411.89667.0d

11. de Villiers EM, Sandstrom RE, zur Hausen H, Buck CE. Presence of papillomavirus sequences in condylomatous lesions of the mamillae and in invasive carcinoma of the breast. Breast Cancer Res. 2005;7(1):R1-11. doi:10.1186/bcr940

12. Doosti M, Bakhshesh M, Zahir ST, Shayestehpour M, Karimi-Zarchi M. Lack of Evidence for a Relationship between High Risk Human Papillomaviruses and Breast Cancer in Iranian Patients. Asian Pac J Cancer Prev. 2016;17(9):4357-61.

13. Duo D, Ghimenti C, Migliora P, Pavanelli MC, Mastracci L, Angeli G. Identification and characterization of human papillomavirus DNA sequences in Italian breast cancer patients by PCR and line probe assay reverse hybridization. Mol Med Rep. 2008;1(5):673-7. doi:10.3892/mmr_00000011

14. Elagali AM, Suliman AA, Altayeb M, Dannoun AI, Parine NR, Sakr HI, Suliman HS, Motawee ME. Human papillomavirus, gene mutation and estrogen and progesterone receptors in breast cancer: a cross-sectional study. Pan Afr Med J. 2021;38:43. doi:10.11604/pamj.2021.38.43.22013

15. Fernandes A, Bianchi G, Feltri AP, Perez M, Correnti M. Presence of human papillomavirus in breast cancer and its association with prognostic factors. Ecancermedicalscience. 2015;9:548. doi:10.3332/ecancer.2015.548

16. Frega A, Lorenzon L, Bononi M, De Cesare A, Ciardi A, Lombardi D, Assorgi C, Gentile M, Moscarini M, Torrisi MR, French D. Evaluation of E6 and E7 mRNA expression in HPV DNA positive breast cancer. Eur J Gynaecol Oncol. 2012;33(2):164-7.

17. Fu L, Wang D, Shah W, Wang Y, Zhang G, He J. Association of human papillomavirus type 58 with breast cancer in Shaanxi province of China. J Med Virol. 2015;87(6):1034-40. doi:10.1002/jmv.24142

18. Gannon OM, Antonsson A, Milevskiy M, Brown MA, Saunders NA, Bennett IC. No association between HPV positive breast cancer and expression of human papilloma viral transcripts. Sci Rep. 2015;5(1):18081. doi:10.1038/srep18081

19. Ghaffari H, Nafissi N, Hashemi-Bahremani M, Alebouyeh MR, Tavakoli A, Javanmard D, Bokharaei-Salim F, Mortazavi HS, Monavari SH. Molecular prevalence of human papillomavirus infection among Iranian women with breast cancer. Breast Dis. 2018;37(4):207-13. doi:10.3233/BD-180333

20. Glenn WK, Heng B, Delprado W, Iacopetta B, Whitaker NJ, Lawson JS. Epstein-Barr virus, human papillomavirus and mouse mammary tumour virus as multiple viruses in breast cancer. PLoS One. 2012;7(11):e48788. doi:10.1371/journal.pone.0048788

21. Gupta I, Jabeen A, Al-Sarraf R, Farghaly H, Vranic S, Sultan AA, Al Moustafa AE, Al-Thawadi H. The co-presence of high-risk human papillomaviruses and Epstein-Barr virus is linked with tumor grade and stage in Qatari women with breast cancer. Hum Vaccin Immunother. 2021;17(4):982-9. doi:10.1080/21645515.2020.1802977

22. Habyarimana T, Attaleb M, Mazarati JB, Bakri Y, El Mzibri M. Detection of human papillomavirus DNA in tumors from Rwandese breast cancer patients. Breast Cancer. 2018;25(2):127-33. doi:10.1007/s12282-018-0831-2

23. Heng B, Glenn WK, Ye Y, Tran B, Delprado W, Lutze-Mann L, Whitaker NJ, Lawson JS. Human papilloma virus is associated with breast cancer. Br J Cancer. 2009;101(8):1345-50. doi:10.1038/sj.bjc.6605282

24. Hennig EM, Suo Z, Thoresen S, Holm R, Kvinnsland S, Nesland JM. Human papillomavirus 16 in breast cancer of women treated for high grade cervical intraepithelial neoplasia (CIN III). Breast Cancer Res Treat. 1999;53(2):121-35. doi:10.1023/a:1006162609420

25. Herrera-Goepfert R, Khan NA, Koriyama C, Akiba S, Perez-Sanchez VM. High-risk human papillomavirus in mammary gland carcinomas and non-neoplastic tissues of Mexican women: no evidence supporting a cause and effect relationship. Breast. 2011;20(2):184-9. doi:10.1016/j.breast.2010.11.006

26. Herrera-Goepfert R, Vela-Chavez T, Carrillo-Garcia A, Lizano-Soberon M, Amador-Molina A, Onate-Ocana LF, Hallmann RS. High-risk human papillomavirus (HPV) DNA sequences in metaplastic breast carcinomas of Mexican women. BMC Cancer. 2013;13:445. doi:10.1186/1471-2407-13-445

27. Hong L, Tang S. Does HPV 16/18 infection affect p53 expression in invasive ductal carcinoma? An experimental study. Pak J Med Sci. 2014;30(4):789-92. doi:10.12669/pjms.304.4534

28. Islam S, Dasgupta H, Roychowdhury A, Bhattacharya R, Mukherjee N, Roy A, Mandal GK, Alam N, Biswas J, Mandal S, Roychoudhury S, Panda CK. Study of association and molecular analysis of human papillomavirus in breast cancer of Indian patients: Clinical and prognostic implication. PLoS One. 2017;12(2):e0172760. doi:10.1371/journal.pone.0172760

29. Khan NA, Castillo A, Koriyama C, Kijima Y, Umekita Y, Ohi Y, Higashi M, Sagara Y, Yoshinaka H, Tsuji T, Natsugoe S, Douchi T, Eizuru Y, Akiba S. Human papillomavirus detected in female breast carcinomas in Japan. Br J Cancer. 2008;99(3):408-14. doi:10.1038/sj.bjc.6604502

30. Khodabandehlou N, Mostafaei S, Etemadi A, Ghasemi A, Payandeh M, Hadifar S, Norooznezhad AH, Kazemnejad A, Moghoofei M. Human papilloma virus and breast cancer: the role of inflammation and viral expressed proteins. BMC Cancer. 2019;19(1):61. doi:10.1186/s12885-019-5286-0

31. Kroupis C, Markou A, Vourlidis N, Dionyssiou-Asteriou A, Lianidou ES. Presence of high-risk human papillomavirus sequences in breast cancer tissues and association with histopathological characteristics. Clin Biochem. 2006;39(7):727-31. doi:10.1016/j.clinbiochem.2006.03.005

32. Lawson JS, Glenn WK, Salyakina D, Clay R, Delprado W, Cheerala B, Tran DD, Ngan CC, Miyauchi S, Karim M, Antonsson A, Whitaker NJ. Human Papilloma Virus Identification in Breast Cancer Patients with Previous Cervical Neoplasia. Front Oncol. 2015;5:298. doi:10.3389/fonc.2015.00298

33. Li N, Bi X, Zhang Y, Zhao P, Zheng T, Dai M. Human papillomavirus infection and sporadic breast carcinoma risk: a meta-analysis. Breast Cancer Res Treat. 2011;126(2):515-20. doi:10.1007/s10549-010-1128-0

34. Liang W, Wang J, Wang C, Lv Y, Gao H, Zhang K, Liu H, Feng J, Wang L, Ma R. Detection of high-risk human papillomaviruses in fresh breast cancer samples using the hybrid capture 2 assay. J Med Virol. 2013;85(12):2087-92. doi:10.1002/jmv.23703

35. Maldonado-Rodriguez E, Hernandez-Barrales M, Reyes-Lopez A, Godina-Gonzalez S, Gallegos-Flores PI, Esparza-Ibarra EL, Gonzalez-Curiel IE, Aguayo-Rojas J, Lopez-Saucedo A, Mendoza-Almanza G, Ayala-Lujan JL. Presence of Human Papillomavirus DNA in Malignant Neoplasia and Non-Malignant Breast Disease. Curr Issues Mol Biol. 2022;44(8):3648-65. doi:10.3390/cimb44080250

36. Manzouri L, Salehi R, Shariatpanahi S, Rezaie P. Prevalence of human papilloma virus among women with breast cancer since 2005-2009 in Isfahan. Adv Biomed Res. 2014;3:75. doi:10.4103/2277-9175.125873

37. Mareti E, Vavoulidis E, Papanastasiou A, Maretis T, Tsampazis N, Margioula-Siarkou C, Chatzinikolaou F, Giasari S, Nasioutziki M, Daniilidis A, Zepiridis L, Dinas K. Evaluating the potential role of human papilloma virus infection in breast carcinogenesis via real-time polymerase chain reaction analyzes of breast fine needle aspiration samples from Greek patients. Diagn Cytopathol. 2023;51(7):414-22. doi:10.1002/dc.25130

38. Mendizabal-Ruiz AP, Morales JA, Ramirez-Jirano LJ, Padilla-Rosas M, Moran-Moguel MC, Montoya-Fuentes H. Low frequency of human papillomavirus DNA in breast cancer tissue. Breast Cancer Res Treat. 2009;114(1):189-94. doi:10.1007/s10549-008-9989-1

39. Nascimento KCG, Sao Marcos BF, Fontes PHB, Isidio BEO, Leao SL, da Silva GRP, Lusson DB, Dos Santos DL, Leal LRS, Espinoza BCF, de Macedo LS, de Franca Neto PL, Silva AJD, Silva Neto JC, Santos VEP, de Freitas AC. HPV Detection in Breast Tumors and Associated Risk Factors in Northeastern Brazil. Cells. 2024;13(13):1132. doi:10.3390/cells13131132

40. Naushad W, Surriya O, Sadia H. Prevalence of EBV, HPV and MMTV in Pakistani breast cancer patients: A possible etiological role of viruses in breast cancer. Infect Genet Evol. 2017;54:230-7. doi:10.1016/j.meegid.2017.07.010

41. Ngamkham J, Karalak A, Chaiwerawattana A, Sornprom A, Thanasutthichai S, Sukarayodhin S, Mus-u-Dee M, Boonmark K, Phansri T, Laochan N. Prevalence of Human Papillomavirus Infection in Breast Cancer Cells from Thai Women. Asian Pac J Cancer Prev. 2017;18(7):1839-45. doi:10.22034/APJCP.2017.18.7.1839

42. Pereira Suarez AL, Lorenzetti MA, Gonzalez Lucano R, Cohen M, Gass H, Martinez Vazquez P, Gonzalez P, Preciado MV, Chabay P. Presence of human papilloma virus in a series of breast carcinoma from Argentina. PLoS One. 2013;8(4):e61613. doi:10.1371/journal.pone.0061613

43. Salman NA, Davies G, Majidy F, Shakir F, Akinrinade H, Perumal D, Ashrafi GH. Association of High Risk Human Papillomavirus and Breast cancer: A UK based Study. Sci Rep. 2017;7(1):43591. doi:10.1038/srep43591

44. Sher G, Salman NA, Kulinski M, Fadel RA, Gupta VK, Anand A, Gehani S, Abayazeed S, Al-Yahri O, Shahid F, Alshaibani S, Hassan S, Chawdhery MZ, Davies G, Dermime S, Uddin S, Ashrafi GH, Junejo K. Prevalence and Type Distribution of High-Risk Human Papillomavirus (HPV) in Breast Cancer: A Qatar Based Study. Cancers. 2020;12(6):1528. doi:10.3390/cancers12061528

45. Sigaroodi A, Nadji SA, Naghshvar F, Nategh R, Emami H, Velayati AA. Human papillomavirus is associated with breast cancer in the north part of Iran. ScientificWorldJournal. 2012;2012:837191. doi:10.1100/2012/837191

46. Tawfeik AM, Mora A, Osman A, Moneer MM, El-Sheikh N, Elrefaei M. Frequency of CD4+ regulatory T cells, CD8+ T cells, and human papilloma virus infection in Egyptian Women with breast cancer. Int J Immunopathol Pharmacol. 2020;34:2058738420966822. doi:10.1177/2058738420966822

47. Wang YW, Zhang K, Zhao S, Lv Y, Zhu J, Liu H, Feng J, Liang W, Ma R, Wang J. HPV Status and Its Correlation with BCL2, p21, p53, Rb, and Survivin Expression in Breast Cancer in a Chinese Population. Biomed Res Int. 2017;2017:6315392. doi:10.1155/2017/6315392

48. Widschwendter A, Brunhuber T, Wiedemair A, Mueller-Holzner E, Marth C. Detection of human papillomavirus DNA in breast cancer of patients with cervical cancer history. J Clin Virol. 2004;31(4):292-7. doi:10.1016/j.jcv.2004.06.009

49. Yu Y, Morimoto T, Sasa M, Okazaki K, Harada Y, Fujiwara T, Irie Y, Takahashi E, Tanigami A, Izumi K. Human papillomavirus type 33 DNA in breast cancer in Chinese. Breast Cancer. 2000;7(1):33-6. doi:10.1007/BF02967185

50. Li J, Ding J, Zhai K. Detection of Human Papillomavirus DNA in Patients with Breast Tumor in China. PLoS One. 2015;10(8):e0136050. doi:10.1371/journal.pone.0136050
